# Digital Proxy of a Bio-Reactor (DIYBOT) combines sensor data and data analytics to improve greywater treatment and wastewater management systems

**DOI:** 10.1038/s41598-020-64789-5

**Published:** 2020-05-15

**Authors:** Eric S. McLamore, Ray Huffaker, Matthew Shupler, Katelyn Ward, Shoumen Palit Austin Datta, M. Katherine Banks, Giorgio Casaburi, Joany Babilonia, Jamie S. Foster

**Affiliations:** 1 Agricultural and Biological Engineering, Institute of Food and Agricultural Sciences, University of Florida, Gainesville, FL 32611 USA; 2 School of Population and Public Health, University of British Columbia, 2206 E Mall Vancouver, BC V6T 1Z3 Canada; 3 MIT Auto-ID Labs, Department of Mechanical Engineering, Massachusetts Institute of Technology, 77 Massachusetts Avenue, Cambridge, MA 02139 USA; 4 MDPnP Interoperability and Cybersecurity Labs, Biomedical Engineering Program, Department of Anesthesiology, Massachusetts General Hospital, Harvard Medical School, 65 Landsdowne Street, Cambridge, MA 02139 USA; 5 NSF Center for Robots and Sensors for Human Well-Being, Purdue University, 156 Knoy Hall, Purdue Polytechnic, West Lafayette, IN 47907 USA; 6 Civil Engineering, Texas A&M University, College Station, TX USA, College Station, TX, 77843 USA; 7 Department of Microbiology and Cell Science, University of Florida, Space Life Science Lab, Merritt Island, FL 32953 USA

**Keywords:** Environmental biotechnology, Chemical engineering

## Abstract

Technologies to treat wastewater in decentralized systems are critical for sustainable development. Bioreactors are suitable for low-energy removal of inorganic and organic compounds, particularly for non-potable applications where a small footprint is required. One of the main problems associated with bioreactor use is sporadic spikes of chemical toxins, including nanoparticles. Here, we describe the development of DIYBOT (Digital Proxy of a Bio-Reactor), which enables remote monitoring of bioreactors and uses the data to inform decisions related to systems management. To test DIYBOT, a household-scale membrane aerated bioreactor with real-time water quality sensors was used to treat household greywater simulant. After reaching steady-state, silver nanoparticles (AgNP) representative of the mixture found in laundry wastewater were injected into the system to represent a chemical contamination. Measurements of carbon metabolism, effluent water quality, biofilm sloughing rate, and microbial diversity were characterized after nanoparticle exposure. Real-time sensor data were analyzed to reconstruct phase-space dynamics and extrapolate a phenomenological digital proxy to evaluate system performance. The management implication of the stable-focus dynamics, reconstructed from observed data, is that the bioreactor self-corrects in response to contamination spikes at AgNP levels below 2.0 mg/L. DIYBOT may help reduce the frequency of human-in-the-loop corrective management actions for wastewater processing.

## Introduction

Surfactants are used globally for a wide variety of purposes, including: food preparation^1^, drug delivery^2^, detergents^3^, foaming agents^4^, dispersants^5^, and as a seed carbon source for biodegradation of heavy metals in contaminated soil^6,7^. One of the most common surfactants is the anionic compound sodium lauryl ether sulfate (SLES). The fate of SLES in the environment has been characterized for a number of scenarios, including both industrial applications^8^ and household wastewater^3^. In household wastewater, SLES is found in greywater (GW) from bathrooms, including bathtubs or showers, but excluding urinals or toilets. GW constitutes up to 70% of the total indoor wastewater and 23% of the total suspended solids per household (by volume)^3^. Decentralized treatment and reuse of GW has been a growing global interest for decades^9–11^, but poses risk of bacterial contamination and buildup of salts/surfactants/metals if applied improperly^12^.

### Greywater as a resource

As global freshwater reserves diminish, recovery of wastewater such as GW is becoming increasingly important. Applications of recovered GW include groundwater recharge^13,14^, landscaping^15^, and irrigation of fresh produce^16^. Direct application of GW has been shown to result in accumulation of salts and metals in as few as four years^17^. Thus, modern GW recovery strategies suggest partial treatment prior to application. Among the technologies under investigation for on-site treatment of GW in water reuse applications^18^, membrane bioreactors (MBR) potentially provide a low-energy option for removal of salts, surfactants, and particulate matter (see review by Wu^11^).

MBR capacity for household GW treatment is typically 15–30 L/m^2^-hr, with low energy consumption, reduced maintenance demands, and no requirement for addition of exogenous chemicals^19^. Although the stoichiometry of SLES biodegradation is well documented in both anaerobic and aerobic conditions^20^ and MBR mass transport has been characterized in detail^21–23^, variable effluent water quality is often encountered from bioreactors. Previous studies of MBR variability have attributed this low reliability to cake layer fouling, inorganic scaling, or chemical shock^24,25^. Among the emerging chemical contaminants that lead to chemical shock in GW treatment processes, engineered silver nanoparticles (AgNP)^26,27^ are of global concern. AgNP can be found in laundry waste water^28^, kitchen sink waste water^29^, and air conditioner condensate, all sources of GW^30^ that can end up in MBR treatment systems. Recent studies indicated that AgNP undergo various chemical transformations in GW, and the toxicity as well as the environmental fate vary widely^31^.

In bioreactors, AgNP are thought to be trapped by microbe-derived exopolymeric substances (EPS) and rapidly transformed to less soluble/problematic compounds, such as AgS^32–34^. Thuptimdang *et al*.^35^ showed that biofilm age and EPS content are critical factors in AgNP toxicity for biofilms, with humic acid playing an important role in sulfidation of AgNP. Although recent efforts have focused on use of bioreactors to sequester and remove engineered AgNP from wastewater^36^, the biggest deterrent to widespread application is the variable effluent quality and lack of control. There is an urgent need for real-time sensor systems that can be coupled with analytical tools and numeric models for improving control of bioreactors.

### Digital proxy for bioreactor management

The concept of using sensor data to inform bioreactor management has been investigated for decades^37–39^. Realization of this concept requires convergence of bioreactor chemistry, mass transport, real-time sensing, and predictive models; an emerging concept referred to as a digital twin^40,41^. For a digital twin to be a reality, cross-sectional and longitudinal effects of common operational conditions must be well characterized and modeled accurately with statistically robust methods. Only a few studies in the biopharmaceutical industry report attempts to create aspects of a bioreactor digital twin^42–44^. These studies provide guidance on best practices for development of digital twins or digital proxies in other applications such as smart water quality management systems. If applied to the water reuse sector, digital twins have predictive and/or responsive capabilities for real time, dynamic natural resource management. The first step towards a digital twin is to create a digital proxy that blends stoichiometry, mass transport and real-time sensing via a combination of longitudinal and cross-sectional studies.

In this study, we demonstrate the development of DIYBOT (Digital Proxy of a Bio-Reactor), a tool to improve management of small-scale decentralized wastewater systems. To construct the digital proxy, a cross-sectional study was first conducted by collecting data to characterize biological, physical, and chemical parameters during silver nanoparticle (AgNP) dosing at the biofilm scale (100 mL flowcell) and also at the reactor scale (bench-top 5 L reactor) (Supplemental Fig. S1). Next, a longitudinal study was conducted with *in situ* sensors (pH and O_2_) to collect real-time effluent water quality data during continuous operation with intermittent AgNP dosing. *In situ* sensor data were used to reconstruct observed bioreactor phase-space dynamics and extract a phenomenological digital proxy. Phase-space dynamics were tested for dynamic correspondence with observed dynamics to characterize observed reactor performance. DIYBOT can be used to extrapolate and predict bioreactor dynamics beyond the sampled performance data and can be combined with other studies of chemical, physical, and biological behavior to produce a predictive management tool. DIYBOT is the first step towards a digital twin^40^ for dynamic system optimization and/or automation, which is a crucial tool needed for providing decision support and early warning of system upset.

## Results and Discussion

### Imaging and material analysis

**Figure 1:**
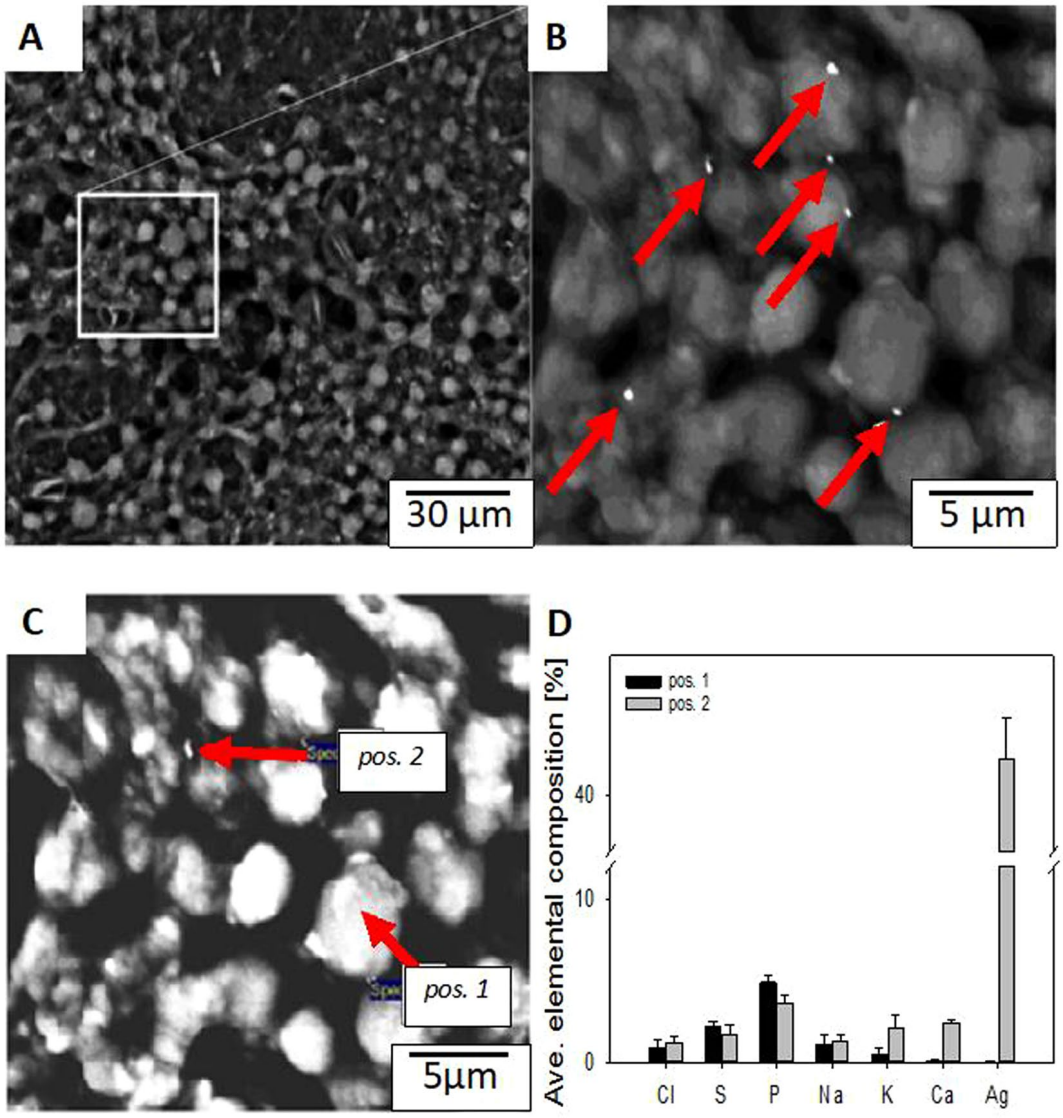
Scanning electron micrographs showing metallic nanoparticles bound to the surface of a HfMBR biofilm. (**A**) Low resolution SEM shows a dense network of cells bound by EPS. (**B**) Inset from panel A indicates small 20 ± 6 nm particles bound to the surface of the biofilm. (**C**) Representative image of HfMBR biofilm with two regions of interest highlighted to indicate location of elemental analysis; position 1 = spectrum acquired on cell surface with no visible nanoparticle, position 2 = spectrum for 25 nm nanoparticle near the surface of a cell. (**D**) Average elemental analysis from 20 spectrum acquired for biofilms without nanoparticles (pos. 1) and with nanoparticles (pos. 2).

Biofilms were extracted from microreactors after 30 days of steady state GW degradation (less than 5% variation in primary substrate degradation), and individual hollow fibers were transferred to flow cells for imaging analysis, material characterization, and biochemical/biophysical characterization. Bright field microscopic imaging of individual fibers showed that the biofilm contained a homogenous distribution of cells and extracellular polysaccharides (EPS), with an average biofilm thickness of 1400 ± 210 μm (Supplemental Fig. S2). The small white structures in Fig. 1 are AgNP (1.0 ppm; particle size = 20–100 nm) that adhered to the surface of the biofilm. Based on scanning electron microscopy (SEM) analysis, the average size of AgNP on the biofilm surface was 245 ± 10 nm (n = 30 particles; Supplemental Fig. S3). AgNP aggregated in discrete regions approximately 20 μm × 20 μm (Fig. 1A), however, there were regions on each sample where no AgNP were visible on the surface (images not shown). From this high-resolution image (Fig. 1B), it is unclear whether the AgNP were surface bound, trapped in EPS near the water interface, or within cells located in the top layer of the biofilm. Previous studies have shown that AgNP can be trapped by EPS in fixed film wastewater treatment reactors, and that AgNP do not penetrate into deep layers within the stratified matrix^45^. However, it is difficult to compare the results of this study with these earlier efforts as the fate of nanoparticles in biofilms can be highly dependent on the specific surface chemistry, size of the AgNP, and the surface chemistry of the biofilm.

Electron dispersive X-ray (EDX) spectroscope analysis (Fig. 1C,D) was performed to confirm nanoparticles (NP) observed on the biofilm surface were indeed from the AgNP mixture. In Fig. 1C, position 1 denotes the location where spectrum was acquired on a cell surface with no visible NP, and position 2 denotes the location where spectrum was acquired near a visible NP. The atomic percent of sulfur and silver was significantly higher for position 2 (near the NP) compared to the isolated cell with no visible NP (position 1). This result confirms that the observed NP in Fig. 1B are from the AgNP solution spiked into the system. The baseline S, Na, and Cl signals present (Fig. 1D) were due to degradation of sulfur-rich SLES, the primary surfactant in the greywater simulant. The NPs on the biofilm surface were likely α-Ag_2_S (Supplemental Fig. S4; Supplemental Table S1), which is similar to other results for AgNP in municipal wastewater biofilms^34,45–47^.

### Biochemical ion flux after AgNP exposure

**Figure 2:**
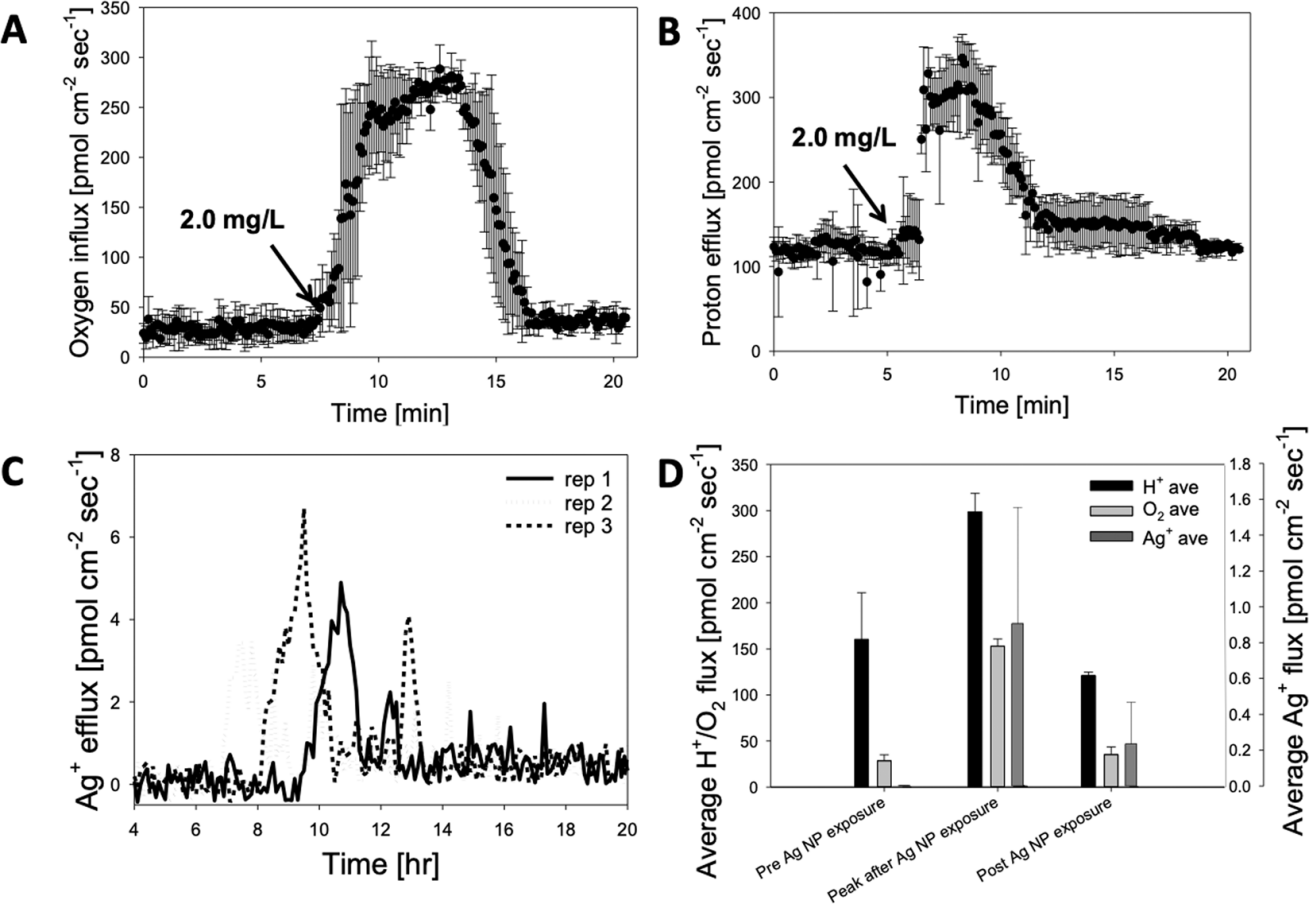
Mechanistic study relating biochemical and physical parameters after AgNP exposure for a single (isolated) hollow fiber in a 500 mL microreactor. Time series showing real-time (**A**) O_2_ influx and (**B**) H^+^ efflux. (**C**) Average H^+^ and O_2_ flux before, during, and after exposure to AgNP (n = 3 replicates). (**D**) Detachment rate during AgNP shock under constant fluid shear. Detachment rate increase linearly with AgNP concentration. The recovery detachment rate increases when the AgNP concentration is 3.0 mg/L.

After confirming that AgNP adhere to GW-degrading biofilms, biochemical flux and detachment (sloughing) were analyzed for individual fibers using previously published methods^48–50^. The baseline O_2_ flux (Fig. 2A) was 29 ± 7 pmol cm^−2^ sec^−1^, and the baseline H^+^ flux (Fig. 2B) was 160 ± 50 pmol cm^−2^ sec^−1^, which is similar to previous measurements for hollow fiber membrane aerated bioreactors (HfMBR) biofilms^49,50^. Prior to addition of AgNP, there was no detectable Ag^+^ near the biofilm surface and the flux (0.02 ± 0.03 pmol cm^−2^ sec^−1^) was approximately zero (Fig. 2C). Addition of AgNP caused a rapid burst in H^+^ efflux and O_2_ influx; H^+^ efflux increased by approximately 300% within 90 seconds (peak H^+^ efflux was 299 ± 20 pmol cm^−2^ sec^−1^), whereas O_2_ influx increased by 500% within 5 min (e.g. peak O_2_ influx was 153 ± 8 pmol cm^−2^ sec^−1^). Ag^+^ efflux near the biofilm surface increased to as high as 6.0 pmol cm^−2^ sec^−1^ within two minutes of AgNP addition. After 15 to 20 min, H^+^, O_2_ and Ag^+^ flux returned to within 5% of baseline levels for AgNP pulse additions of 1 mg/L, referred to as quasi-steady state (QSS).

The average QSS H^+^ efflux after AgNP addition (Fig. 2D; 121 ± 48 pmol cm^−2^ sec^−1^) was not significantly different than pre-exposure levels; this outcome was also the case for O_2_ influx after AgNP exposure (35 ± 8 pmol cm^−2^ sec^−1^). However, the post exposure Ag^+^ flux (0.35 ± 0.33 pmol cm^−2^ sec^−1^) was significantly higher than pre-exposure levels (p < 0.001, α = 0.05). Since sulfidation rate of AgNP is dependent on size^45,47^, it was expected that the Ag^+^ release rate would be much higher from these relatively large NPs (100–375 nm). Although we did not conduct a detailed kinetic study on the sulfidation of AgNP, it is likely that the excessive sulfur liberated from SLES degradation enhanced the sulfidation of aggregated AgNP to silver sulfide (Ag_2_S) for these relatively large NP.

The pattern of rapid H^+^/O_2_ flux followed by a slow recovery is similar to previous work with HfMBR biofilms investigating oxidative stress response systems^49^. This sub-lethal stress response system has been well characterized in a wide variety of environmental conditions. For example, Gu *et al*.^51^ showed that AgNP cause severe oxidative stress during long-term exposure (i.e., 22 days) of flocculent sludge to AgNP. Additionally, Mallevre *et al*.^52^ showed that monoculture *Pseudomonas putida* biofilms exposed to a single pulse of AgNP (10 mg/L) recovered within 24 h of exposure, which is similar to the results shown here. In monoculture *Azotobacter vinelandii*, Zhang *et al*.^53^ showed that the magnitude of oxidative stress may be dependent on AgNP size, but this has not been confirmed in mixed culture biofilms. Although the specific stress response pathway for GW degrading bacteria exposed to AgNP is unknown, this microsensor flux data gives insight into the underlying mechanisms and suggests that efflux pumping and oxidative bursts may be a component of the response to AgNP exposure.

### Biofilm detachment after AgNP exposure

**Figure 3:**
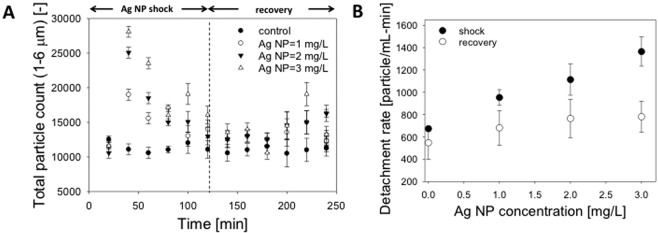
Detachment of biofilms exposed to Ag NP under constant fluid shear. (**A**) Continuous monitoring of detached particles from the hollow fiber biofilm flowcell (**B**) Average detachment rate during shock and recovery period shows decreased detachment correlates with increasing Ag NP concentration.

Biofilm detachment was investigated as a sub-lethal stress response mechanism due to AgNP exposure using previously established methods^54,55^. This study involved both individual fibers in flowcells and bench scale 0.5 L reactors. Prior to AgNP exposure, biofilm (1 to 6 μm mean particle diameter) detached at a constant rate of 600 ± 129 particles mL^−1^ min^−1^ under constant fluid flow, which is similar to Huang *et al*.^54^ (Fig. 3A). Within 20 min of adding AgNP to the flowcell, there was a significant increase in the number of detached particles, which reached a peak after approximately 50 min and declined to near pre-exposure levels; noted as “shock” in Fig. 3. The number of detached particles measured 120 to 250 min after addition of AgNP (noted as “recovery” in Fig. 3) was significantly higher for a concentration of 3.0 AgNP than all other tests (p < 0.0001, α = 0.05); however, there was no significant differences for the other test conditions. There was a concentration-dependent increase in the total detachment of biofilm aggregates for all AgNP concentrations tested for data associated with the “shock” period, but no change for data associated with the recovery period (Fig. 3B). This dose-dependent response followed by a recovery to baseline levels is similar to previous studies of detachment due to sub lethal chemical exposure^56^.

To confirm the results of the individual hollow fiber study, biomass was collected from a HfMBR sump every 30 days after the AgNP dosing experiments. Similar to the study of individual fibers, reactors exposed to AgNP showed a significantly higher total solid content for AgNP concentrations of 3.0 mg/L, but no significant difference at lower NP concentrations (Supplemental Fig. S5). These results are important for understanding the fate of AgNP in dynamic systems, such as fixed film reactors and limiting release of AgNP or Ag^+^ into the environment. Gu *et al*.^51^ showed that AgNP had a detrimental effect on flocculent sludge, primarily by reducing the ammonia oxidation potential of the community. AgNP concentrations in sludge amended soils are estimated to be 662 ng/kg on an annual basis, and the silver compounds are primarily found within the top 0–30 cm of soil where exposure to germinating seeds and nitrifying bacteria is likely^57^. Future studies of detachment and AgNP/Ag^+^ fate are needed as some taxa, such as *Nitrosomonas* activity, are known to be inhibited by AgNP^58,59^ and there is a well-documented mechanism for mitochondria inhibition by AgNP^60,61^.

### HfMBR treatment capacity after AgNP exposure

**Figure 4:**
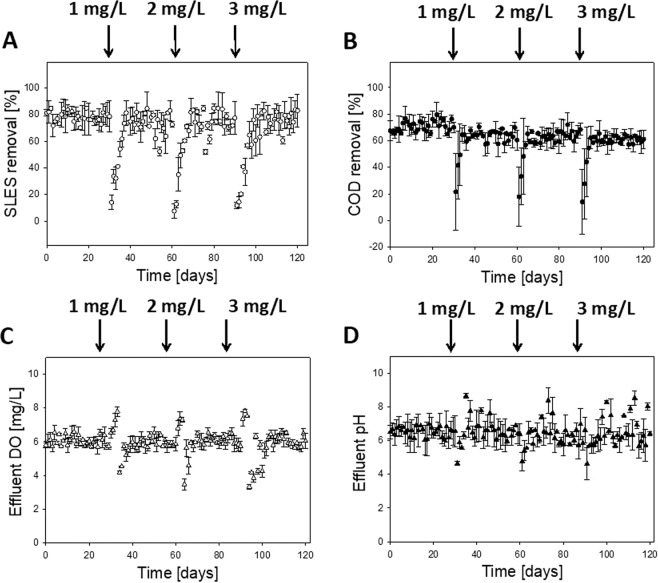
Reactor effluent quality for 5 L bench scale HfMBR reactors exposed to cumulative AgNP pulse additions. After 30 days of baseline steady state performance, 1.0 mg/L of AgNP was added (20–100 nm mixture), followed by an additional pulse (total of 2.0 mg/L) at day 60 and another pulse at day 90; all additions marked by vertical arrows. (**A)** Average percent SLES removal measured by LCMS. (**B**) Average percent COD removal. (**C**) Average effluent DO. (**D**) Average effluent pH. Error bars represent standard deviation of the arithmetic mean (n = 3 reactors).

HfMBR effluent water quality was assessed by measuring percent chemical oxygen demand (COD) removal, SLES removal, and effluent pH, dissolved oxygen (DO), and Ag^+^ concentration for grab samples taken from 0.5 L bench scale reactors (raw data shown in supplemental section). After inoculation, reactors were monitored for 30 days of steady state effluent water quality was observed (note: steady state was defined as <5% change in water quality parameters measured). At steady state, the baseline COD and SLES removal were 70.9 ± 4.1% and 78.2 ± 3.8%, respectively, whereas the average effluent pH and DO were 6.5 ± 0.3 and 6.1 ± 0.4 mg-DO/L, respectively (Fig. 4). Addition of AgNP (1.0 mg/L) caused a temporary decrease in effluent water quality, followed by a recovery to QSS. Average COD removal, SLES removal, and effluent pH each decreased significantly and then recovered, while effluent DO increased followed by a recovery to 3.0 mg-DO/L. After addition of 1.0 mg/L AgNP, the steady state COD and SLES removal were significantly lower than baseline levels (p < 0.001, α = 0.05), but effluent pH and DO were not statistically different. After recovering to steady state for at least five days, another AgNP pulse added (cumulative concentration of AgNP was 2.0 and 3.0 mg/L).

The response for each concentration of AgNP was similar, although the QSS levels did not significantly change relative to 1.0 mg/L AgNP for any parameter measured. The time associated with recovery to steady state varied for each parameter; in general COD removal and effluent pH recovered quickly within 3.6 hydraulic retention times (HRT), followed by recovery of SLES removal (7.3 HRT) and finally effluent DO (8.2 HRT). The trends were magnified for magnified for higher concentration of AgNP, with recovery lasting from as 5 to 11 HRT (Supplemental Table S2). These results confirm the individual fiber studies (Figs. 2,3) showing that pulse additions of AgNP cause a temporary shock, but the biofilm recovers treatment capacity. Taken together, our results show that the mechanisms associated with the temporary shock likely involves ion efflux mechanisms, as well as active detachment mechanisms comparable to those first discovered by Bott and Love^62^. This general pattern has also been shown for sequencing batch reactors exposed to AgNP^36^, where a short-term (e.g. 4 day) decrease in COD removal was followed by a recovery to baseline levels within one solids retention time.

The average effluent Ag^+^ concentration in bench top HfMBR was monitored to validate the individual fiber study. Effluent Ag^+^ (Supplemental Fig. S6) increased with cumulative exposure to increasing concentrations but returned to baseline levels within 4–5 days (note: the highest level was 101 ± 83 μg Ag^+^/L after addition of 3.0 mg AgNP/L). We hypothesize that this return to baseline levels was due to sulfidation of AgNP, leading to the formation of insoluble Ag_2_S. The insoluble Ag_2_S was bound to biofilm EPS, resulting in a decrease in the free Ag^+^ measured in the reactor effluent. Typically, there are two routes for sulfidation of AgNP to form Ag_2_S nanoparticles; namely the indirect route (i.e., oxidative dissolution to Ag^+^ followed by precipitation) and the indirect route (i.e., solid-fluid heterogeneous reaction)^95^. Since both O_2_ and Ag^+^ were present in the effluent, it is likely that Ag_2_S NP were formed by a combination of the direct route and indirect route proposed by Liu *et al*.^63^. In addition to the likelihood for Ag_2_S NP formation, biofilm detachment noted in Fig. 3 may have contributed to release of Ag^+^ and/or AgNP prior to sulfidation of AgNP to Ag_2_S NP.

### Impact of AgNP on bioreactor microbial diversity

**Figure 5:**
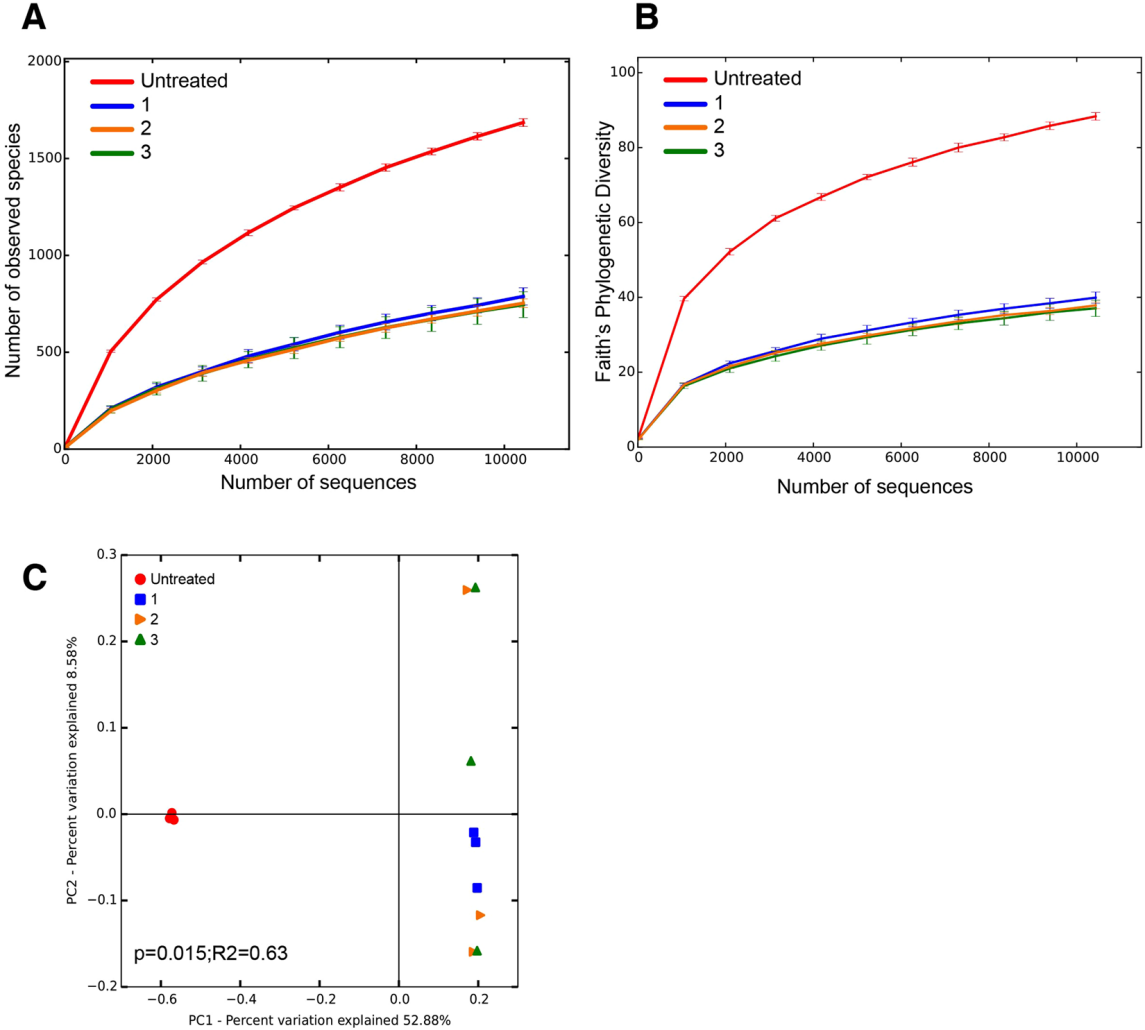
Overview of the negative impact of AgNP on the microbial diversity of bioreactor communities. Rarefraction curves depicting the (**A**) number of observed species and (**B**) Faith’s phylogenetic index of the different bioreactor treatments revealing a dramatic decrease in taxa upon exposure to AgNP in a range of concentrations (1.0 mg/L; 2.0 mg/L; and 3.0 mg/L). (**C**) PCoA plot comparing the different sequencing replicates of the bioreactor treatments revealing that the majority of the differences observed between the microbial population is the due to the presence of AgNP.

To assess how the AgNP potentially impact the microbial diversity of the bioreactor community, three 16S rRNA gene amplicon libraries were generated for each of the untreated and AgNP-treated bioreactors resulting in a total of 174,184 raw bacterial sequences (n = 12 libraries). After quality filtering using the Quantitative Insights Into Microbial Ecology (QIIME) tool, 162,818 high-quality filtered reads were recovered with a mean of 13,568 reads per replicate (Table 1). There was little variation between the three replicates for each treatment indicating a high level of reproducibility between biological replicates. The open-reference Operational Taxonomic Units (OTU) picking approach in QIIME identified 4,397 OTUs associated with 36 phyla, however, only 12 phyla had a relative abundance higher than 0.5%. The microbial diversity was the highest in the untreated bioreactors with 2,458 recovered unique OTUs compared to 1529–1609 OTUs in the AgNP-treated reactors (Table 1; Fig. 5). Community richness (i.e., alpha diversity) was assessed using rarefaction curves normalized at 10,431 sequences per sample and the number of observed species (Fig. 5A) and Faith’s phylogenetic diversity index (Fig. 5B) revealed that the untreated bioreactor samples showed a significantly higher diversity compared to those AgNP-treated bioreactors. These results suggest that the AgNP exposure decreased the overall diversity of the microbial communities (Fig. 5C).

**Table 1:** Bacterial 16S rRNA amplicon diversity analysis of bioreactor samples treated with AgNPs.

	Untreated Bioreactor	Bioreactor 1	Bioreactor 2	Bioreactor 3
Sequences (post filtering)	40923	40336	37462	37452
Rarefraction depth^a^	10431	10431	10431	10431
OTUs (97% similarity)	2458	1609	1529	1544
OTUs ≥ 1%	7	15	15	15
Chao (mean ± SEM^b^)	1758 ± 31	999 ± 50	922 ± 35	902 ± 61
Faith’s Phylogenetic Index (mean ± SEM)	64 ± 0.8	28 ± 1.0	27 ± 0.6	26 ± 1.4
Sequence length	271–371	271–361	271–365	271–366
Base pair average	304	298	297	295

^a^Number of randomized sequences used to generate diversity measures reflect the lowest numbers of sequences recovered from one of the bioreactor replicates. ^b^Standard error of the mean.

**Figure 6:**
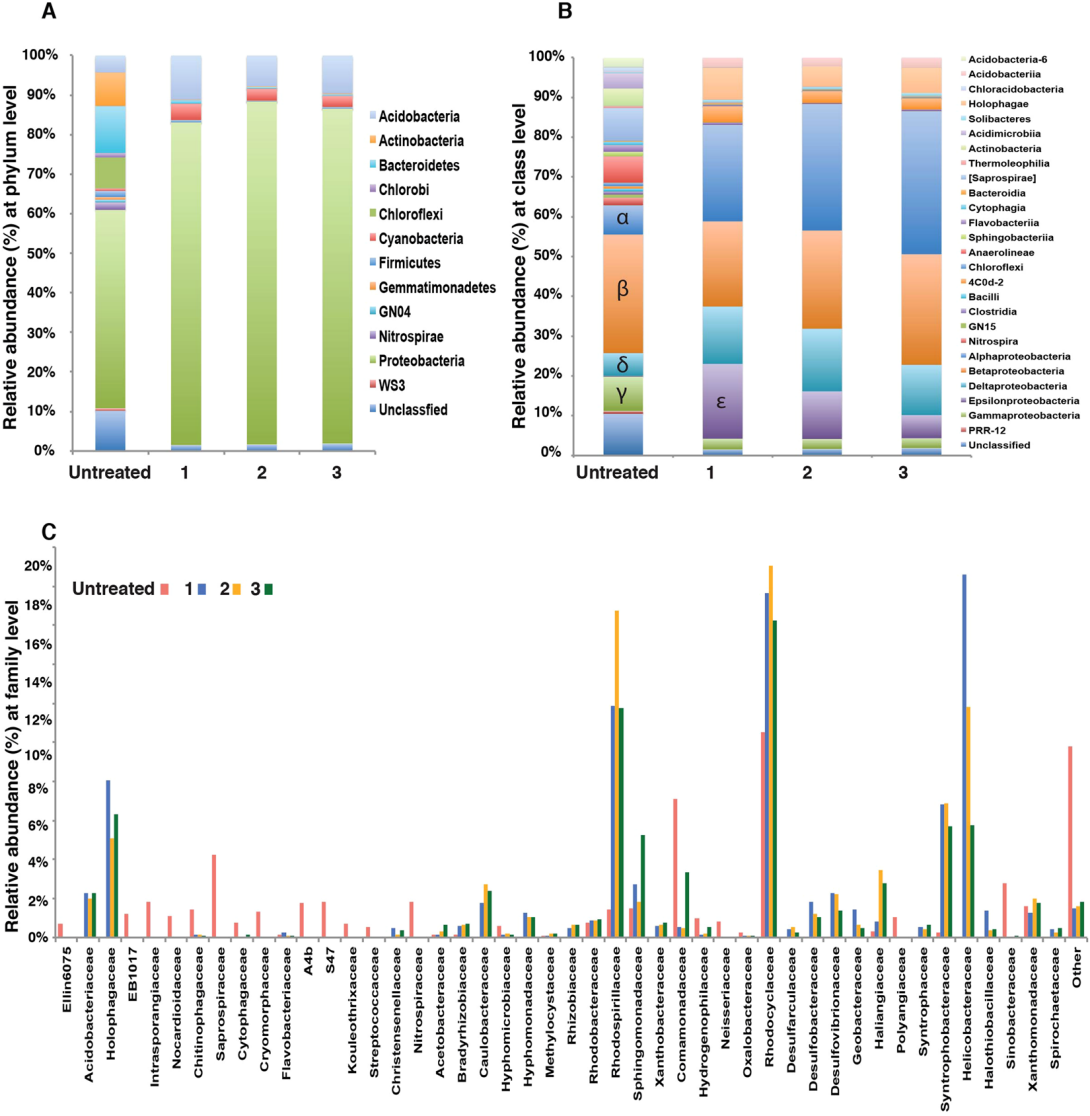
Overview of the microbial diversity of bioreactors treated with AgNP. Relative abundance of taxa associated with untreated and treated reactors at the (**A**) phyla, (**B**) class and (**C**) family levels.

Within the untreated samples the Proteobacteria dominated comprising 48% of the population (Fig. 6A). Bacteroidetes (11.5%), Actinobacteria (8.2%) and Chloroflexi (7.5%) were also in high relative abundance within the untreated bioreactors. Upon AgNP treatment the relative abundances of the community shifted and many of the taxa present in the untreated samples, such as the Chlorobi, Chloroflexi, Gemmatimonadetes, GN04, Nitrospirae, and WS3, were undetectable in AgNP-treated biofilms. Additionally, the Actinobacteria and Bacteroidetes also declined, decreasing to less than 1% in the AgNP-treated biofilms. Although most taxa were negatively impacted by AgNP treatment there were two phyla that showed increases when exposed to the AgNP including the Proteobacteria, which rose to between 81–86% of the total population post-treatment and the non-photosynthetic cyanobacteria-like 4C0d-2, which increased from 0.65% of the population to 2.8–3.1% of the AgNP treated samples (Fig. 6B).

Although the overall proteobacterial population increased in the bioreactors exposed to AgNP, there were distinct shifts within the different classes. In the Alphaproteobacteria there was a pronounced increase in the purple non-sulfur Rhodospirillaceae from 1.42% in the untreated 11.8 to 16.8% in the AgNP-treated bioreactors as well as a moderate increase in the Sphingomonadaceae (1.8–5.2%). A similar increase of Sphingomonadaceae in the presence of Ti-NP and ZnO-NP have been observed in soil communities exposed for 60 days^64^. Additionally, there was an increase in Betaproteobacteria family Rhodocyclaceae from 10.5% of population in the untreated reactors to 16.2–19.1% in the AgNP-treated reactors. The Rhodocyclaceae family includes many denitrifying bacteria that typically occupy aquatic and waste water habitats^65^. Within the Deltaproteobacteria there was a slight increase in the Desulfobacterales and Desulfovibrionales, which harbor several sulfate-reducing taxa.

There were few differences between the different AgNP treatments suggesting that microbial community is highly sensitive to the presence of AgNP even at lower dosages. Although one family of Epsilonproteobacteria, the Helicobacteraeceae, known to harbor several human pathogens, was highly represented in the AgNP Bioreactor 1 treatment, the relative abundance of that taxa decreased as the AgNP concentration increased. A principal coordinate analysis (PCoA) of the unweighted UniFrac rarefied distance matrix showed a distinctive clustering pattern of the different treatments (Fig. 6C). The plot revealed that 52.8% of the differences in the bioreactor treatments could be explained by the presence of the AgNP. Within the treatments there was little variation, with only 8.58% of the differences between the treated samples being explained by the AgNP exposure (P = 0.015; R^2^ = 0.63, adonis). To investigate the link between sensor data and AgNP exposure, we next developed a phenomenological model based on real-time sensor data.

### Phenomenological model of bioreactor dynamics

A phenomenological model was developed based on real-time effluent sensor data recorded at high signal acquisition frequency (100 Hz over the course of 40 days). Sensor data was collected and stored on an external hard drive after 3 days, and sensors were calibrated every 3 days. The first 40 days of data were collected after the HfMBR reached steady state operation (less than 5% deviation in treatment) (Supplemental. Fig. S7). Mean reactor pH and DO during healthy operation were 7.3 ± 0.2 and 178 ± 4 μM, but there were harmonic oscillations within the pH data that had a normal period of 2.1 ± 0.4 min, and the magnitude was 7.3 ± 0.2; reactor DO data also exhibited oscillatory behavior with a normal period of 2.9 ± 0.7 min and a magnitude of 178 ± 4 mM (Supplemental Fig. S8A). Starting on day 40, a pulse of AgNP (1.0 mg/L) was added every 30 days with the reactor under continuous operation. After pulse addition of AgNP, the pH and DO data transitioned into a damped harmonic oscillator, although the timing was not coincident (Supplemental Fig. S8B). For AgNP concentrations of 1.0–2.0 mg/L, the pH and DO data returned to near-baseline levels (within 8%) and resumed characteristic oscillatory behavior (see Table S2, row 2 for baseline effluent conditions). The oscillation periodicity and magnitude were significantly different than baseline values, thus the oscillations are referred to as quasi steady state (QSS) throughout.

**Figure 7:**
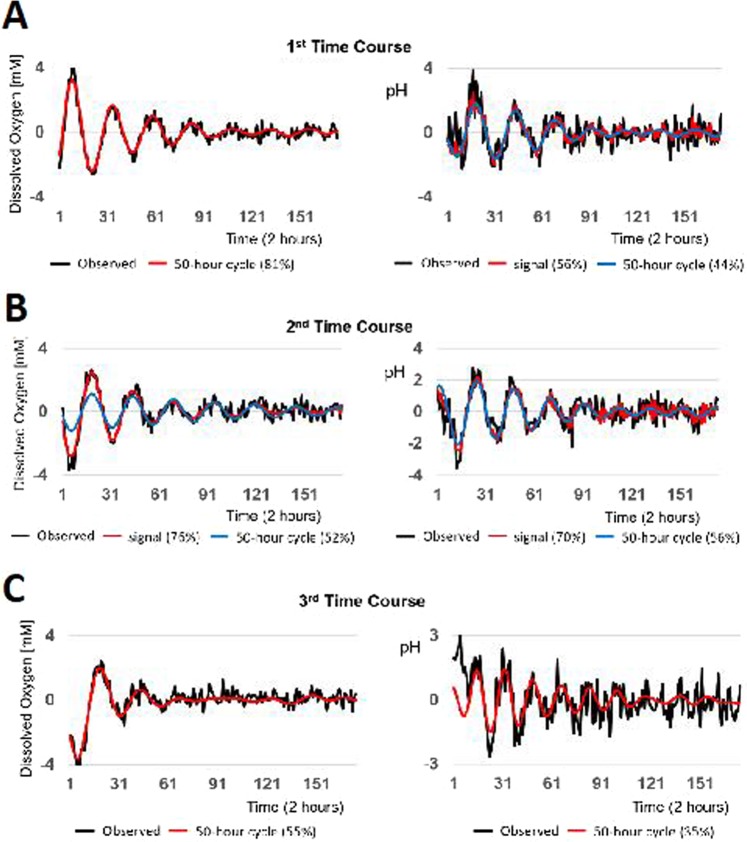
Signal processing of real time O_2_ and pH sensor data using singular spectrum analysis. Measured data are shown in black traces for both O_2_ (left side) and pH (right side), processed signals are shown in red. Time course analysis for (**A**) 1.0 mg/L AgNP, (**B**) 2.0 mg/L AgNP, and (**C**) 3.0 mg/L AgNP.

To investigate the dynamics of response to AgNP in more depth, signal processing of O_2_ and pH time series were analyzed with singular spectrum analysis^66^. Each time course corresponds to an addition of AgNP as shown in Fig. 7. Processed signals for O_2_ and pH (red curves) isolated from the measured records (black curves) were dominated by low-frequency 50-hour cycles composed of 25 two-hour blocks (blue curves in Fig. 7). Signal strengths (reported as percentages) were strong, accounting for a substantial portion of total variation in the corresponding measured records. Following addition of AgNP at all concentrations tested, signal oscillations dampened substantially over time consistent with spiral sink dynamics. Signal processing results were consistent across each subsequent addition with the signal strength varying slightly for each pulse AgNP addition. To further investigate the observed spiral-sink dynamics, time series were reconstructed in phase space dynamics to search for causal interactions between O_2_ and pH data during exposure to AgNP (Supplemental Figs. S9–10).

**Figure 8:**
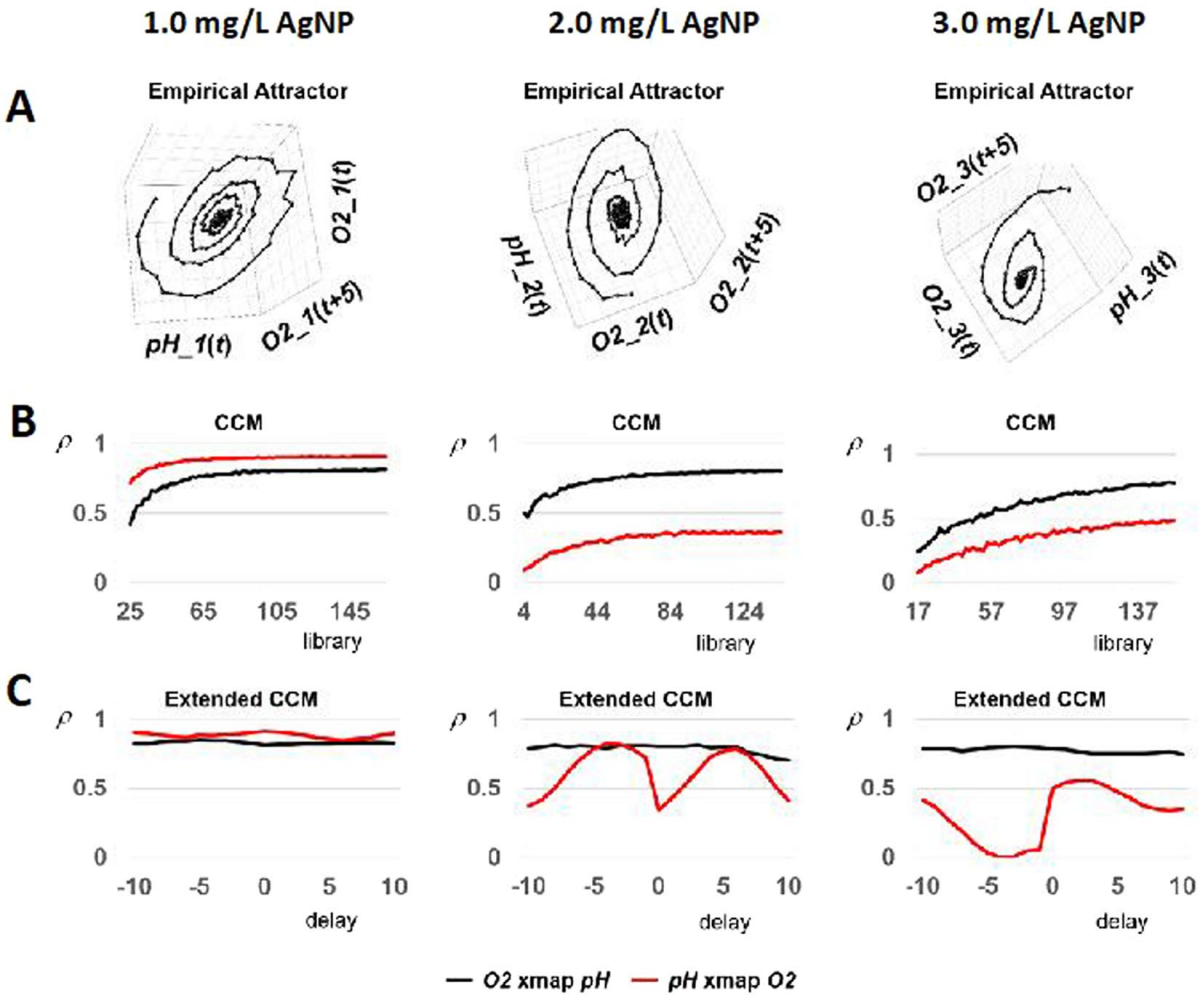
Phase space dynamics of O_2_ and pH time series data for determining causal interactions at varying AgNP concentration. (**A**) Reconstruction of empirical attractors by applying time-delay embedding. (**B**) Convergent cross mapping (CCM) to determine if reconstructed attractors indicate interactions between O_2_ and pH. (**C**) Extended CCM used to rule out false positives of causal interaction.

Empirical attractors were reconstructed by applying time-delay embedding (Fig. 8A). These attractors exhibited three-dimensional spiral-sink dynamics for each AgNP addition using coordinates of: i) real-time (current) values of dissolved oxygen, denoted as O_2_(t); ii) dissolved oxygen values with a delay of five two-hour blocks, denoted as O_2_ (t + 5); and iii) current values of pH, denoted as pH(t). Reconstructed attractors were used to test whether O_2_ and pH interact using convergent cross mapping (CCM)^67^. CCM indicates that pH and O_2_ are strongly coupled when AgNP concentration is 1.0 mg/L since both CCM curves approach relatively high correlation coefficients (ρ) exceeding 0.8 (Fig. 8B). CCM also indicates that pH remains a strong driver of O2 as AgNP increases in concentration since CCM curves continue to approach relatively high correlation coefficients in the neighborhood of 0.8 (black curves). However, when the AgNP concentration is 2.0 mg/L or greater, CCM analysis indicates that O_2_ weakens as a driver of pH since CCM curves approach relatively low correlation coefficients below 0.5 (red curves). To further investigate this phenomena, extended CCM^68^ was applied, which enables ruling out false positives representing synchronized behavior to an outside force rather than true causal interaction (Fig. 8C). The curves indicate true causality by demonstrating peaks in the CCM map and inferring true causality by the delay associated with the peak. Figure 8C indicates that O_2_ is a false driver of pH for 3.0 mg/L AgNP (the noted peak occurs at a positive delay of 3 periods). Taken together, the results in Fig. 8 indicate that O_2_ and pH have strong bi-causal interaction at or below 1.0 mg/L AgNP, this bi-causal interaction is weakened for AgNP concentrations of 2.0 mg/L, and when the AgNP concentration is 3.0 mg/L a unidirectional interaction occurs with pH driving O_2_.

From a biochemical standpoint, interactions between effluent pH and DO are linked to the metabolic transport of pH/O_2_ during ATP synthesis. The digital signature produced by the phenomenological model (pH as a weak driver of O_2_) is most likely a result of oxidative phosphorylation (OxPho) as described in our previous work^49^. We also showed in this previous study that uncouplers of OxPho result in rapid proton efflux that precedes a burst in oxygen influx after exposure to uncoupling agents. Dakal provide a comprehensive review indicating that the mechanistic basis of AgNP antimicrobial affects is most likely oxidative stress^69^, and Bondarenko *et al*.^70^ confirm this by showing that AgNP are localized near the plasma membrane, leaching Ag^+^ to the nearby inner membrane (the site of ATP production).

**Figure 9:**
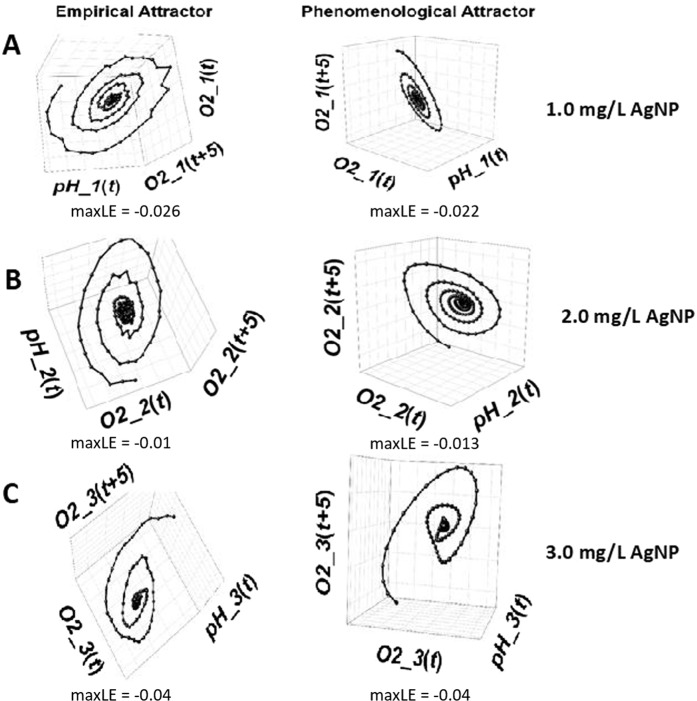
Phenomenological modeling of empirical attractors for AgNP concentration of (**A**) 1.0 mg/L, (**B**) 2.0 mg/L, and (**C**) 3.0 mg/L. Ordinary differential equations, equilibrium and eigenvalues are shown in the Supplemental section.

A phenomenological model was developed by establishing the governing ordinary differential equations (ODE) that describe reactor behavior using the real-time O_2_ and pH data based on Brunton *et al*.^71^. For each concentration of AgNP, the phenomenological attractors reproduced the dynamics characterizing the empirical data, which was characterized by a stable three-dimensional spiral-sink attractor. Figure 9 depicts the combination of a negative real eigenvalue and a conjugate complex pair of eigenvalues with negative real parts (ordinary differential equations (ODE) and eigenvalues are shown in Supplemental Fig. S11–13). Under all conditions, the empirical plots (left panels in Fig. 9) bear remarkable resemblance to the corresponding attractor reproduced by the phenomenological model (right panels in Fig. 9).

Dynamic correspondence between empirical and phenomenological attractors was more rigorously established by comparing maximum Lyapunov exponents measuring the average rate at which infinitesimally-close trajectories on attractors exponentially diverge or converge over time^72,73^. Computational algorithms—such as R routine lyap_k(tseriesChaos) developed by Di Narzo^74^ conventionally generate a semilogarithmic plot of the logarithm of average separation against time periods. The slope of a linear portion of the plot provides an estimate of the maximum Lyapunov exponent. For the attractors shown in Fig. 9, computed values are substantially the same across corresponding empirical and phenomenological attractors (rows in Fig. 9), providing evidence of dynamic correspondence.

**Figure 10:**
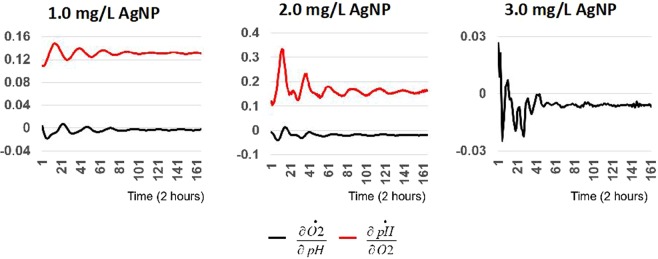
Quantifying the causal interactions of phenomenological modeling with CCM at concentrations of (**A**) 1.0 AgNP mg/L, (**B**) 2.0 AgNP mg/L, and (**C**) A) 3.0 AgNP mg/L.

Causal interactions of the phenomenological ODE models were quantified using CCM and compared to empirical data. During exposure to AgNP concentrations at or below 2.0 mg/L, pH (the system driver) has a negative marginal impact on O_2_ (black curves in Fig. 10A,B), while O_2_ (as the driver) has a positive marginal impact on the pH (red curves in Fig. 10A,B). However, the impact of pH on O_2_ decays to zero as time progresses, indicating that the stress response mechanism was stabilized and reached a new quasi-equilibrium after two hours. After exposure to 3.0 mg/L AgNP (Fig. 10C), pH drives O_2_ response and the system reached a new quasi-equilibrium after approximately two hours. The corresponding partial derivative shows that after AgNP exposure, pH has a detrimental impact on O_2_ and this relationship decays with time.

Sub-lethal stress response in biofilm systems is complex and involves a number of biochemical and biophysical interactions. Table 2 provides a summary of the biochemical and biophysical responses of HfMBR to AgNP at concentrations of 1.0 to 3.0 mg/L. Within 5 min of exposure to AgNP at concentrations of at least 2.0 mg/L, microscale H^+^ efflux and O_2_ influx showed a burst response which returned to QSS levels that were not statistically different than pre-exposure levels. The pattern for 3.0 mg/L AgNP was similar, but the steady state was significantly different than baseline. Biofilm detachment and reactor treatment capacity followed this same trend, with the effect more pronounced at higher AgNP concentrations. Causal correlation showed that after exposure to AgNP at concentrations of at least 2.0 mg/L, pH has a negative short-term impact on O_2_, but this decays to QSS within two hours. For concentrations of 3.0 mg/L AgNP, O_2_ is a weak driver of pH and the interaction decayed to QSS within two hours. Taxa shifts, on the other hand occurred for all concentrations of AgNP.

**Table 2:** Summary of cross-sectional and longitudinal study of HfMBR biofilms during pulse AgNP exposure.

AgNP [mg/L]	H^+^/O_2_ flux^b^	Biofilm Detachment^c^	Treatment Capacity	Causal Correlation^d^	Microbial Diversity
1.0	Burst increase in flux followed by return to qSS^a^ within 2 hours (*Supp*.)	Burst detachment followed by return to qSS within 2 hours (Fig. 3)	Metabolism and reactor pH/O_2_ burst change, followed by return to qSS within 2 hours (Fig. 4)	Proton transport has negative short-term impact on O_2_; decays within 2 hours (Figs. 7,8**)**	Taxa shifts for AgNP> 1.0 mg/L (Figs. 5,6)
2.0	Burst increase in flux followed by return to qSS within 2 hours (Fig. 2)	Burst detachment followed by return to qSS within 2 hours (Fig. 3)	Metabolism and reactor pH/O_2_ burst change, followed by return to qSS within 2 hours (Fig. 4)	pH has negative short term impact on O_2_; decays within 2 hours (Figs. 7,8**)**	Taxa shifts for AgNP> 1.0 mg/L (Figs. 5,6)
3.0	Burst increase in flux. Mean flux decreased over time, but did not return to qSS (*Supp*.)	Burst detachment. Mean values decreased over time, but did not return to qSS (Fig. 3)	Metabolism and pH/O_2_ burst change. Mean values did not return to qSS (*Supp*.)	O_2_ is a weak driver of pH (Figs. 7,8**)**	Taxa shifts for AgNP> 1.0 mg/L (Figs. 5,6)

^a^qSS = quasi steady state, not different than mean levels prior to exposure (p < 0.01, α = 0.05). ^b^Proton efflux and oxygen influx measured at the surface of HfMBR biofilms using microelectrodes. ^c^Particle detachment measured using coulter counter with constant fluid flow. ^d^Causal correlation determined via analysis of phase space dynamics.

### Digital Proxy of a Bio-Reactor – DIYBOT

The series of ODE developed in Figs. 9–10 demonstrate a phenomenological model that was able to capture complex dynamic interactions between high resolution pH and O_2_ real-time data. DIYBOT reproduced the dynamic responses shown in Figs. 7,8, thus the series of ODE can be used as a real-time proxy for bioreactor status. DIYBOT simulated the observed stable-focus dynamics during sub lethal chemical toxicity due to AgNP. The management implications of the stable-focus dynamics reconstructed from observed data are that the bioreactor self-corrects in response to contamination spikes, thus eliminating corrective management strategies faster than 3.6 HRT recovery period. In future efforts, DIYBOT could be used to extrapolate and predict bioreactor dynamics beyond the sampled performance data and develop a predictive tool that is coupled with real-time sensors integrated with auto-updated analytics.

Although not studied in detail here, the predictive capacity of DIYBOT is an important step towards a digital twin approach^40^. Digital twins provide four important features, including: 1) improved process understanding; 2) high temporal response; 3) enhanced automation; and 4) development of embedded, distributed, or modular control units^75^. To our knowledge, DIYBOT is a first step towards a digital twin for wastewater treatment reactors, and may have applications in terrestrial systems (as demonstrated in this study) as well as autonomous systems, such as long duration space exploration^22,76^.

The results presented here establish the DIYBOT platform for development of smart water treatment systems, which could be reproduced for a myriad of real-world applications. Here, we focused on the test case of nanoparticle-dosing from laundry wastewater as an example of a bioreactor system shock, but the approach could be used in many other applications relevant to bioreactor management. One caveat, however, is that the DIYBOT proxy must be developed for the specific stressor of interest and under conditions that are similar to the operational environment, and additional water quality analysis is needed (particularly in the longitudinal study). Phenomenological models with high process specificity have been developed for xanthum gum bioreactors^77^, cellulose hydrolysis^78^ soli substrate fermentation bioreactors^79^ and wine fermentation^80^, among others. These examples, therefore, represent important future targets for the development of DIYBOT functional capabilities but must be extended to include additional modeling capabilities, perhaps including agent-based systems to impart functions such as continuous updates. Without including aspects of continuous model update (and perhaps aspects of machine learning), the system is restrained to a “proxy” as described here. In future studies, development of a true digital twin would include these additional capabilities, with more emphasis placed on prediction and forecasting.

Advances in computer science and data analytics may pave the way for adoption of the DIYBOT concept, but there are currently major challenges in terms of semantic interoperability, standards, and ontologies^40^. If the aforementioned improvements are coupled with recent development of cheaper, smaller, more accurate sensors, real-time automation of wastewater treatment is a possibility in the foreseeable future, leading to the evolution of digital proxies toward the development of digital twins.

## Methods

### Reactor construction and nanoparticle dosing

Hollow fiber membrane aerated bioreactors (HfMBR) were constructed as described in previous literature^54,55,81^. Briefly, reactor shells were constructed using clear PCV pipe and couplings attached to 1 cm thick PVC plates; the bottom coupling contained a chamber for equally distributing gas to the fiber lumen (Supplemental Fig. S14). Two different reactors were used, including microreactors in which there was a total 0.5 L volume, and bench scale reactors containing 5 L in total volume. Each plate contained holes (0.64 cm) with threaded ferrules (Direct Industry, Sarasota, FL). Polydimethyl sulfoxide membranes (0.17 cm outside diameter; 0.08 cm inside diameter; Dow Corning Co., Midland, MI) were fitted through the ferrules on each plate and arranged in straight fiber geometry. The silicon membranes facilitated biofilm growth on the substrate (liquid) side, and provided aeration from the fiber lumen with a transmembrane air pressure of 10kPa^50,54^. The fiber packing density (0.69) and porosity of the reactors (0.78) was the same for each reactor size.

Reactors were inoculated with acclimated sludge taken from the aeration basin of the University of Florida wastewater treatment plant according to Sharvelle *et al*.^82,83^. After inoculation, reactors were fed gray water (GW) simulant on recycle until a homogenous biofilm was visible on the fibers and reactor effluent quality (see below for details) changed by less than 5% (approximately 45 days). After formation of a homogenous biofilm, all reactors were operated at a recirculation ratio of 20:1 and a loading rate of 3.0 L/d with a hydraulic retention time of 1.1 days^49,50,54^. The composition of the GW simulant was based on previous studies^83,84^. SLES, the primary surfactant in the GW, was purchased as STEOL-CS330 (28.8% SLES) from Stepan Co. (Northfield, Illinois), all other compounds for the greywater recipe^85–89^ were purchased from Sigma Aldrich (Atlanta, GA).

For AgNP dosing, a stock solution of citrate capped 20–100 nm AgNP (ACS Material, >99.9% purity) was prepared in phosphate buffered saline (PBS) buffer at room temperature, and sonicated with a horn tip sonicator prior to all studies to limit NP aggregation (Suppplemental Fig. S15). Once the bioreactors were at steady state for 30 days, AgNP (total concentration of 1.0 mg/L) were added to the influent line of each reactor. This process was repeated three times for bench scale reactors and microreactors; cumulative AgNP concentrations of 1.0, 2.0 and 3.0 mg/L were tested. The AgNP concentration range covers expected levels leached from textiles^90^. After AgNP dosing, reactors were placed in a class 2 biohood and dismantled. Fibers with biofilms (approximately 50 g of sample) were immediately immersed in RNAlater (Germantown, MD), and then stored for subsequent microbial diversity analysis.

### Cross-sectional study: single fiber analysis

For imaging and material analysis, individual fibers with biofilms were extracted from microreactors, and fixed in a phosphate buffered solution (PBS) containing 4% glutaraldehyde for 1 h at 23 °C. After fixation the fibers were washed four times in PBS to remove residual media, dried under ambient conditions for 8 h, sputtered with gold, and fixed to carbon tape as described in Jaroch *et al*.^91^. The fibers were visualized using a NOVA NanoSEM high-resolution field emission scanning electron microscope and analyzed using an INCA 250 electron dispersive X-ray (EDX) spectroscope (Oxford Instruments, Oxford, UK). The spectral contribution of carbon was removed prior to analysis due to potential interference from the carbon tape fixative and the atomic percentage of the remaining elements was determined (reported as atomic percent). Zeta potential and dynamic light scattering measurements of AuNP in 5 mM sodium phosphate buffer adjusted to pH 7.4. Both measurements were performed after sonication using a Zetasizer Nano ZS (Malvern Panalytical, Cambridge, UK).

For measurements of oxygen/ion flux and biofilm detachment, individual hollow fibers with intact biofilm were removed from the microreactors via 0.25-in ferrules and transferred to a flow cell according to previous studies^54,55,81^. A fiber optic optrode^92^ was used for measuring oxygen flux, and ion-selective microelectrodes^93,94^ were used for measuring H^+^ or Ag^+^ flux (see noted references for sensor fabrication details). All sensors were calibrated in GW simulant before and after each analysis following the protocols in the referenced literature above (Supplemental Fig. S16). Flux was measured by using computer controlled stepper motors to position microsensors within 2–3 μm of the biofilm surface, and then sensors were oscillated according to McLamore *et al*.^49^. Biofilms were then exposed to GW flow with the same free stream velocity as the microreactor, and allowed to stabilize for 30 min while measuring flux. After 30 min, AgNP were added to the influent line of the flowcell and flux was continuously monitored. Flowcell effluent samples were collected in autoclaved bottles every 5 min based on Zhang *et al*.^55,81^ and analyzed. All flux data was post processed using a finite impulse response filter based on a weighted moving average algorithm as described by McLamore *et al*.^95^.

To measure the biofilm detachment rate from an individual fiber, effluent samples from the flowcell were collected every 5 min according to Zhang *et al*.^55,81^. Samples were analyzed using a MS4 Coulter Counter (Beckman Coulter, Inc., Brea, California) with a 20 μm aperture tube (detection range of 0.4 to 12 μm). Detached particles were diluted to approximately 105 particles per mL with Isoton II solution (Beckman Coulter, Brea, California) based on previous studies^54,55^.

### Cross-sectional study: benchtop reactor analysis

Reactor effluent chemical oxygen demand (COD) and total nitrogen (TN) were measured daily using colorimetric kits (Hach, Loveland, Colorado). Effluent SLES concentration was measured daily using liquid chromatography coupled with mass spectrometry (LC-MS) (Thermo Finnigan, Waltham, Massachusetts) based on Levine *et al*.^96^. An Alltech Alltima C18 column (Nicholasville, Kentucky) was used at a flowrate of 0.3 mL/min with mobile phases of 25 mM ammonium acetate buffer pH 3.6 (mobile phase A) and 100% acetonitrile (mobile phase B). Effluent pH was measured daily using an in line potentiometric probe according to McLamore *et al*.^50^ and Sharvelle *et al*.^83,97^. Effluent oxygen was measured daily with an in-line fiber optic O_2_ sensor fabricated using the methods in McLamore *et al*.^98^. Effluent free silver was measured using a custom Ag^+^-selective electrode fabricated using the methods in McLamore *et al*.^94^.

### Cross-sectional study: microbial diversity and bioinformatics analysis

Three DNA extractions of the bioreactor samples were performed for each replicate (100 mg) using the PowerSoil DNA extraction kit according to manufacturer’s instructions (Qiagen, Carlsbad, CA), normalized, and pooled. DNA from each replicate was then PCR-amplified using a 454-fusion primer that targeted the V1–2 region of the 16S rRNA gene and contained a distinct oligonucleotide barcode tag (Supplemental Table S3)^99^. The PCR reagents and conditions for the amplicon library preparation were completed as previously described^100^. The 16S rRNA gene amplicon libraries were sequenced using the 454 GS-FLX platform with Titanium chemistry (Roche, Branford, CT) at the University of Florida’s Interdisciplinary Center for Biotechnology Research. The raw sequence data files were deposited into the NCBI sequencing read archive under number PRJNA378777.

The 16S rRNA barcoded amplicon sequences were analyzed using the Quantitative Insights Into Microbial Ecology (QIIME v. 1.9.1.) pipeline^101^. Sequences were quality-filtered and demultiplexed using default parameters and the filtered reads were assigned to operational taxonomic units (OTUs) at 97% identity using an open-reference OTU picking approach using UCLUST v1.2.22q^102^ against the Greengenes database (v. 13_8^103^;). Taxonomic classification was performed with UCLUST for each OTU and the sequences were aligned using PyNAST v1.2.2^101^ against the Greengenes core set^104^. Based on this alignment a phylogenetic tree was built using FastTree v2.1.8^105^. Rarefaction curves were computed using two different metrics: the Faith’s Phylogenetic Diversity (PD) richness estimator^106^ and the observed species metric, which reported the number of different bacterial OTUs at a rarefaction depth of 10,431 sequences/sample. Principal coordinates analyses (PCoA) were generated from UniFrac unweighted distance matrices^107^. A nonparametric test and the adonis^108^ method were used to determine the statistical significance related to the diversity analyses.

### Longitudinal study: real-time pH and DO measurements

For real-time monitoring of reactor pH and DO, probes were inserted into sampling ports within 2 cm of the effluent withdraw port. The pH probes were purchased from DF Robot (Beijing, China), and all pH data at a sample frequency of 100 Hz using a National Instruments acquisition described in our previous work^93^. Fiber optic DO probes were purchased from Presens (Microx4, Sarasota, FL). The tip of the DO probe was replaced at least once per month following the procedures in McLamore *et al*.^109^. All probes were removed from sampling ports and calibrated at least once per week. Data were stored on an external hard drive until analyzed.

### Longitudinal study: phenomenological modeling

Signal processing of O_2_ and pH sensor data recorded at 100 Hz acquisition frequency were analyzed using Singular Spectrum Analysis^66^. A sequence of methods adapted from nonlinear time series (NLTS) analysis^72,73^ was used to reconstruct deterministic system dynamics from observed output without prior knowledge of model equations. The logic flow for NLTS used here involved detection, reconstruction, and modeling of bioreactor dynamics from observed output data (pH and O_2_) (Supplemental Fig. S17). Singular spectrum analysis method of signal processing was used to separate structural variation (signal) from unstructured variation (noise) in the performance records, and the strength of isolated signals was then measured according to Golyandina *et al*.^110^. Strong signals were then tested for nonlinear stationarity using the nonlinear cross prediction method^111^. Bioreactor dynamics from strong stationary output signals was reconstructed with phase space reconstruction^112^. Surrogate data testing^113^ was then used to analyze the statistical likelihood that apparent structure in reconstructed phase space was due to deterministic nonlinear dynamics (as opposed to random forcing).

The results of this analysis were used as an indicator of whether or not reconstructed bioreactor dynamics were dissipative (i.e., long-term evolution of system dynamics bounded within a subset of phase space) using established NLTS analysis protocol^72,73^. The NLTS threshold of dissipative behavior criteria was used to determine whether long-term system dynamics could be modeled with relatively few degrees of freedom - regardless of the complexity or dimensionality of the original system. Reconstructed dissipative dynamics were used to test for conjectured causal interactions among observed sensor variables with convergent cross mapping^67^. Finally, a set of ordinary differential equations (phenomenological models) was derived from output signals to numerically simulate reconstructed bioreactor dynamics. This model has predictive capacity to quantify and characterize detected causal interactions over time and space using with methods established by Brunton *et al*.^71^.

Reconstructed attractors were used to test whether O_2_ and pH interact with a two-step procedure. First, we applied Convergent Cross Mapping (CCM), which detects causality running from Y (i.e., the potential driver) to X (i.e., the response variable) if the empirical attractor reconstructed from X can be used to cross map values of Y, CCM curves detect variables as a strong driver of system dynamics if the correlation coefficient (ρ) exceeds 0.8. Second, we applied Extended CCM^68^ to rule out false positives representing synchronized behavior to an outside force rather than true causal interaction. This test imposes backward and forward delayed responses between the driver and response variables, and finds true causality when the CCM curve reaches a peak at a negative delay. A model composed of ordinary differential equations (ODE) derived from the measured O_2_ and pH time series was developed based on Brunton *et al*.^71^. Finally, the causal interactions of this model were quantified by computing partial derivatives based on the series of ODE to determine how incremental increases in the driver variable impacts the response variable.

## Supplementary information

Supplementary information.

## Data Availability

The datasets generated during and/or analyzed during the current study are available from the corresponding author on reasonable request. The raw sequence data files were deposited into the NCBI sequencing read archive under number PRJNA378777.
