# Supplementary material for: Digital Proxy of a Bio-Reactor (DIYBOT) combines sensor data and data analytics to improve greywater treatment and wastewater management systems
Source: Sci Rep. 2020 May 15;10:8015. doi: 10.1038/s41598-020-64789-5 (PMC7229150; doi:10.1038/s41598-020-64789-5)
Supplement: Supplementary file 1 — Supplementary information. [file 41598_2020_64789_MOESM1_ESM.pdf]

Supplemental Materials:

Digital Proxy of a Bio-Reactor (DIYBOT) combines sensor data and data analytics to improve greywater treatment and wastewater management systems

Eric S. McLamore[1*], R. Huffaker[1], Matthew Shupler[1], Katelyn Ward[1], Shoumen Palit Austin Datta[1,2,3,4], M. Katherine Banks[5], Giorgio Casaburi[6], Joany Babilonia[6], Jamie S. Foster[6]

[1] Agricultural and Biological Engineering, Institute of Food and Agricultural Sciences, University of Florida, Gainesville, FL 32611

[2] MIT Auto-ID Labs, Department of Mechanical Engineering, Massachusetts Institute of Technology, 77 Massachusetts Avenue, Cambridge, MA 02139

[3] MDPnP Interoperability and Cybersecurity Labs, Biomedical Engineering Program, Department of Anesthesiology, Massachusetts General Hospital, Harvard Medical School, 65 Landsdowne Street, Cambridge, MA 02139

[4] NSF Center for Robots and Sensors for Human Well-Being, Purdue University, 156 Knoy Hall, Purdue Polytechnic, West Lafayette, IN 47907

[5]Civil Engineering, Texas A&M University, College Station, TX

College Station, TX 77843

[6]Department of Microbiology and Cell Science, University of Florida, Space Life Science Lab, Merritt Island, FL 32953

* Corresponding author

Element	Biofilm surface prior to AgNP exposure	NP aggreages on bioilm 30 days after AgNP exposure
O	60.0 ± 4.5	27.5 ± 4.0
S	25.3 ± 3.2	42.2 ± 3.1
Cl	14.6 ± 2.6	0.2 ± 0.2
Ag	0.1 ± 0.2	20.1 ± 2.9

Table S1. Average elemental analysis determined by EDX before and after AgNP exposure.

SNP [ppm]	Percent COD Removal	Percent SLES removal	Effluent pH	Average DO [mg-DO/L]	Effluent Ag+ [μg-Ag_+/L]
0	70.9±4.1	78.2±3.8	6.5±0.3	6.1±0.4	14±9
1.3	61.8±4.6*	70.9±6.0*	6.5±0.6	6.0±0.5	36±30
2.6	61.6±5.2*	71.9±5.6*	6.4±0.7	6.2±0.5	58±35*
3.9	60.9±2.3*	74.8±7.2*	6.7±0.5	5.9±0.6	101±83*

Table S2. Average effluent data after addition of AgNP. Asterisk denotes significant difference compared to baseline levels (t-test; α=0.05). Influent concentrations for the greywater were as follows: COD= 290 ± 25 mg-COD/L;

SLES= mg/L; pH= ; DO= mg/L; and Ag+= 0.0 mg/L.
he average chemical oxygen demand (COD), dissolved organic carbon (DOC), total nitrogen, and ammonium (NH4+) concentrations of the liquid-phase waste stream were,
14.3 -+0.5mMDOC,
6.9-+2.2mMN,and
0.4 ± 0.2 mM N. The mixed surfactant solution contributed 90 -+ 1% (14.5 ± 0.1 mM COD) of the total COD.

Sample Name	454 primer[b]	Barcode[c]	16S primer[d]	Specificity
27F	B	none	AGAGTTTGATCCTGGCTCAG	*Bacteria*
Untreated-338R-rep1	A	CCCGCCAT	TGCTGCCTCCCGTAGGAGT	universal
Untreated-338R-rep2	A	CCTACCGC	TGCTGCCTCCCGTAGGAGT	universal
Untreated-338R-rep3	A	AAGCAACG	TGCTGCCTCCCGTAGGAGT	universal
ConcA-338R-rep1	A	CCAACCTT	TGCTGCCTCCCGTAGGAGT	universal
ConcA-338R-rep2	A	GGAATTGG	TGCTGCCTCCCGTAGGAGT	universal
ConcA-338R-rep3	A	AACCAACC	TGCTGCCTCCCGTAGGAGT	universal
ConcB-338R-rep1	A	TTAAGGCC	TGCTGCCTCCCGTAGGAGT	universal
ConcB-338R-rep2	A	CCGGCCTT	TGCTGCCTCCCGTAGGAGT	universal
ConcB-338R-rep3	A	AAGGCCTT	TGCTGCCTCCCGTAGGAGT	universal
ConcC-338R-rep1	A	AACGAAGC	TGCTGCCTCCCGTAGGAGT	universal
ConcC-338R-rep2	A	TTCGAAGC	TGCTGCCTCCCGTAGGAGT	universal
ConcC-338R-rep3	A	AATACCGC	TGCTGCCTCCCGTAGGAGT	universal

Table S3. Primers used for generating 16S rRNA barcoded library. [a]Number denotes the location on the *Escherichia coli* 16S rRNA gene. [b]454 Life Sciences sequencing primer A (GCCTTGCCAGCCCGCTCAGCT) and primer B (GCCTCCCTCGCGCCATCAG) with a TA linker preceding the 16S rRNA gene primer. [c]Barcodes taken from Hamady et al., 2008, end with a CA linker preceding the 16S rRNA gene primer. [d]Reference for 16S rRNA gene primer taken from Suzuki et al., 1996.

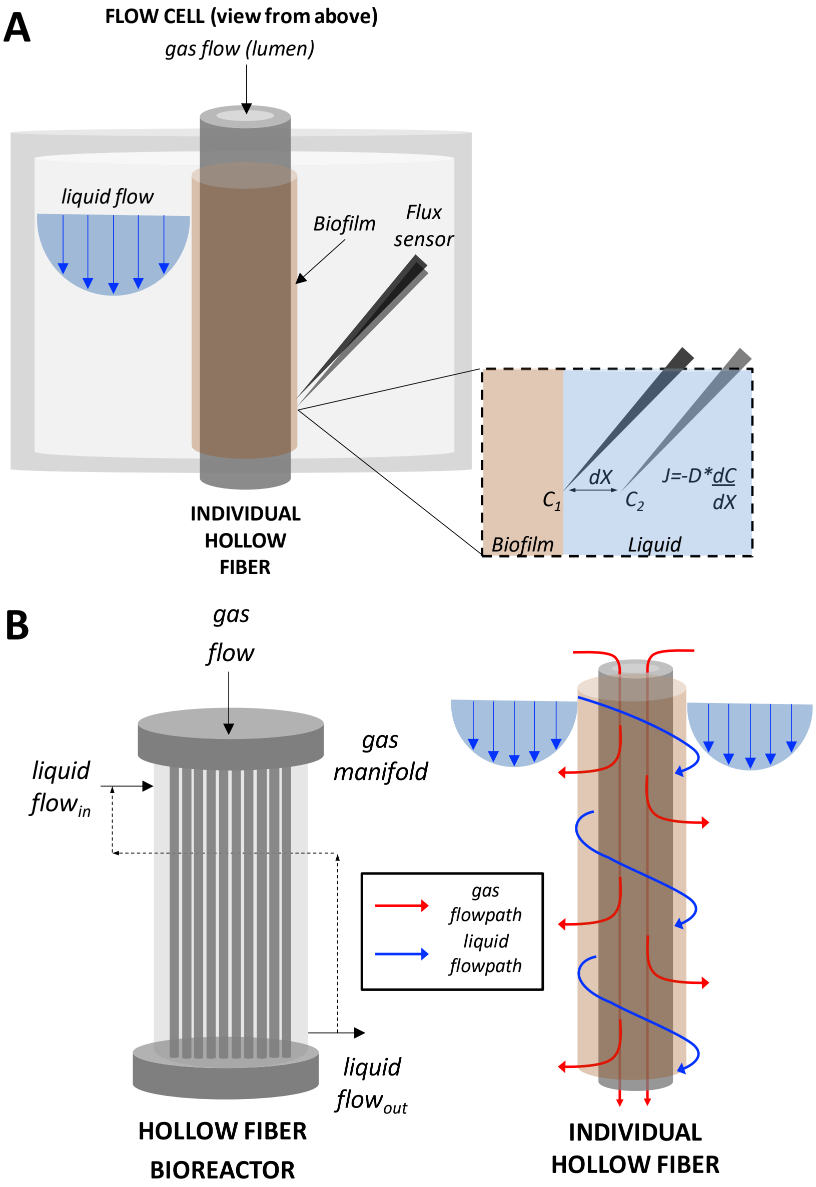

Figure S1. Schematic depicting overview of **A)** Cross sectional study of biofilm physiology conducted in flow cells (microelectrode study shown as an example). **B)** Longitudinal study conducted in bench HfMBR with two phase flow.

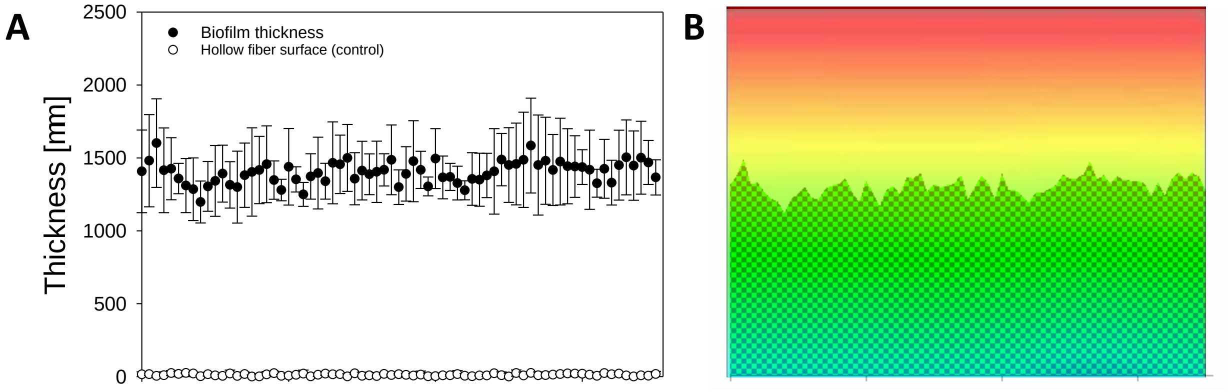

Figure S2. **A)** Biofilm thickness for individual fibers extracted from HfMBR measured using the methods in McLamore et al[2]. Average biofilm thickness was 1400 ± 210 µm. Error bars represent standard deviation of the arithmetic mean. **B)** Free stream velocity near individual fiber modeled with COMSOL according to McLamore et al[2]. The plot is overlaid with the thickness measurement from panel A.

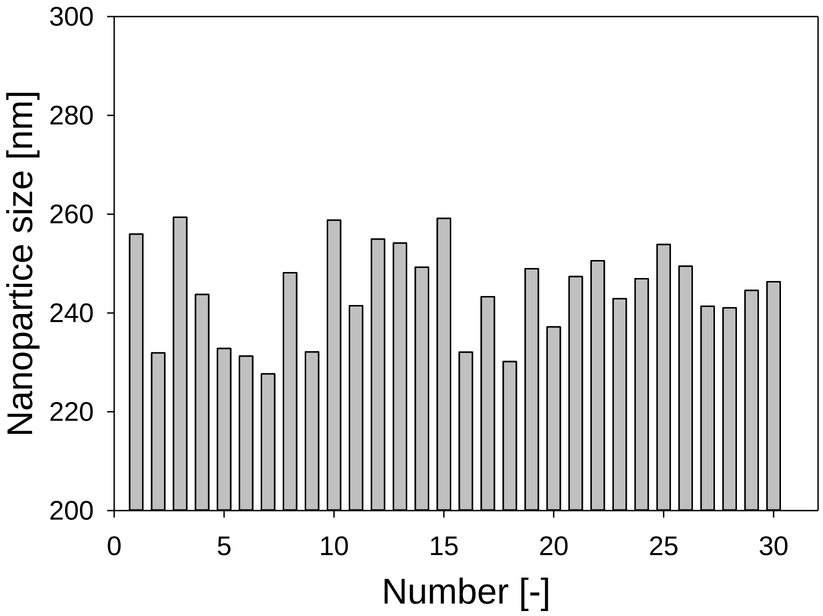

Figure S3. Average AgNP size on biofilm surface estimated from SEM images. Particles on the biofilm surface was 245 ± 10 nm (n = 30 particles).

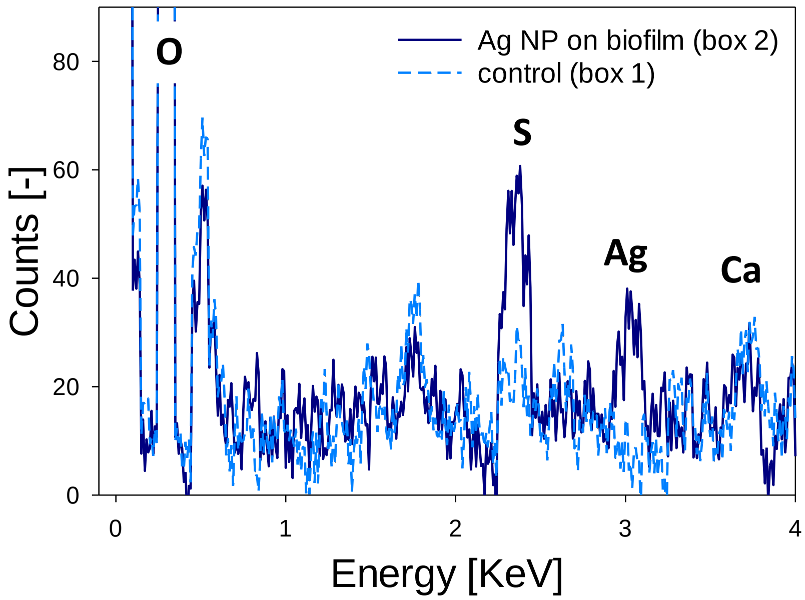

Figure S4. Elemental analysis of biofilm shown in Fig 1C. The plot indicates that the NPs imaged on the biofilm surface were likely α-Ag_2S due to the relatively high peaks near 2.3 KeV indicating sulfur groups (compared to control with no AgNP).

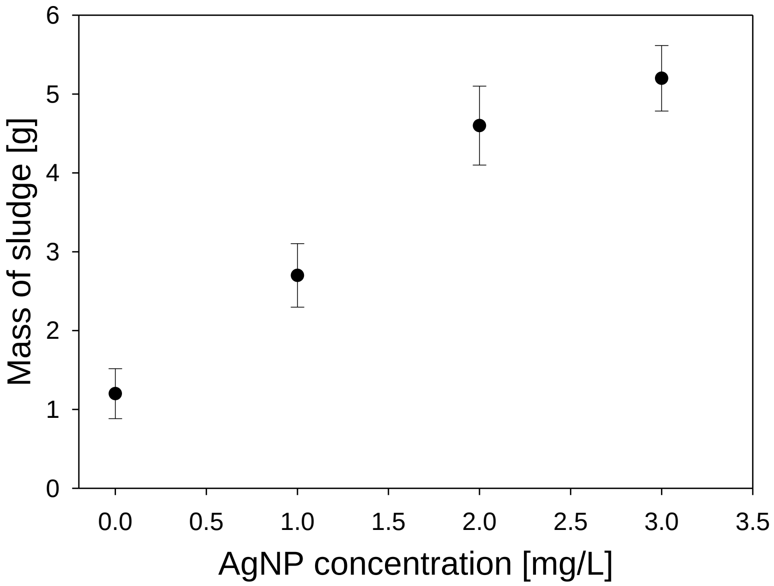

Figure S5. Mass of sludge in the reactor sump, measured 30 days after addition of AgNP. Error bars represent the standard deviation of the arithmetic mean (3 replicate reactors).

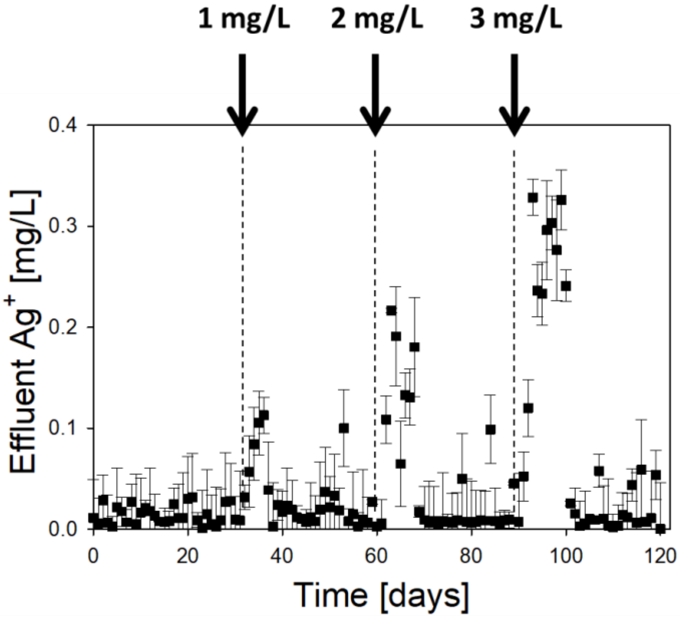

Figure S6. Ionic silver (Ag+) concentration measured in effluent of 5L bench scale HfMBR reactors exposed to AgNP. Concentration of AgNP is indicated by the vertical arrow.

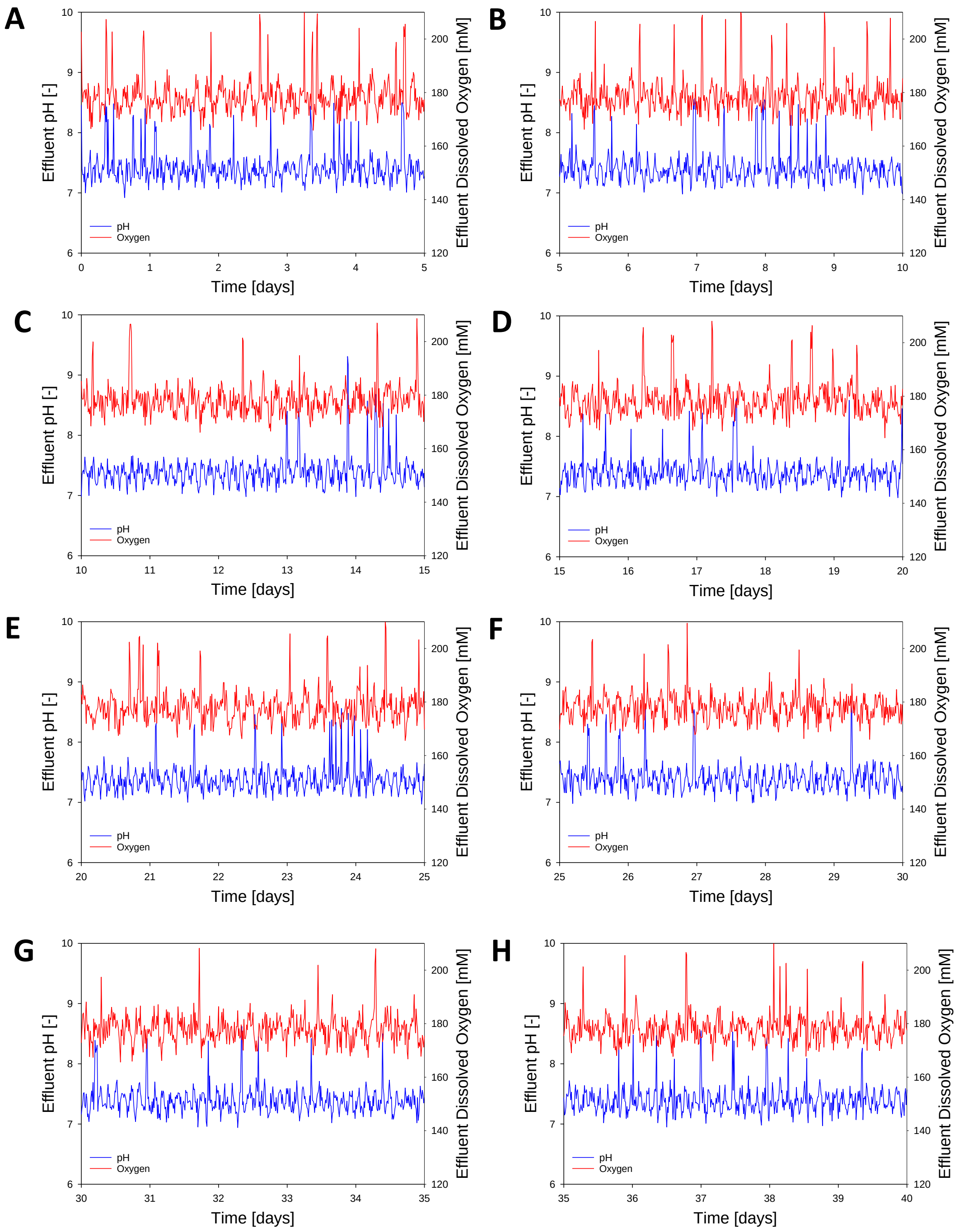

Figure S7. Effluent pH and DO data recorded continuously during steady state operation (no AgNP pulse additions). The data is separated by 5 hour monitoring period in each panel.

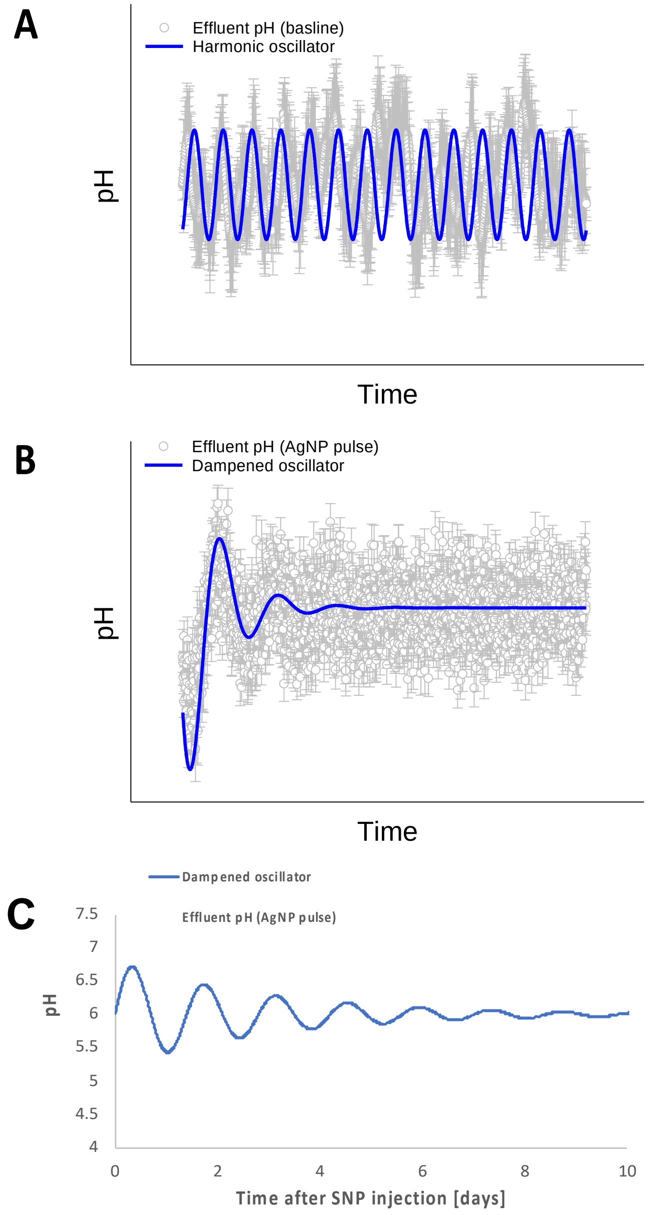

Figure S8. Empirical modeling of reactor data before and after AgNP pulse. **A)** Prior to AgNP addition, the real time data shows regular harmonic oscillations which were empirically modeled using Chi2 fitting. **B)** After addition of AgNP, the real time data shows damped oscillations which were empirically modeled using Chi2 fitting. The time value on the x-axis in panel A-B is normalized to pulse injection and shown as dimensionless for comparison of all replicate data. **C)** Representative example of the damped harmonic oscillator after addition of AgNP for one replicate test (in triplicate)

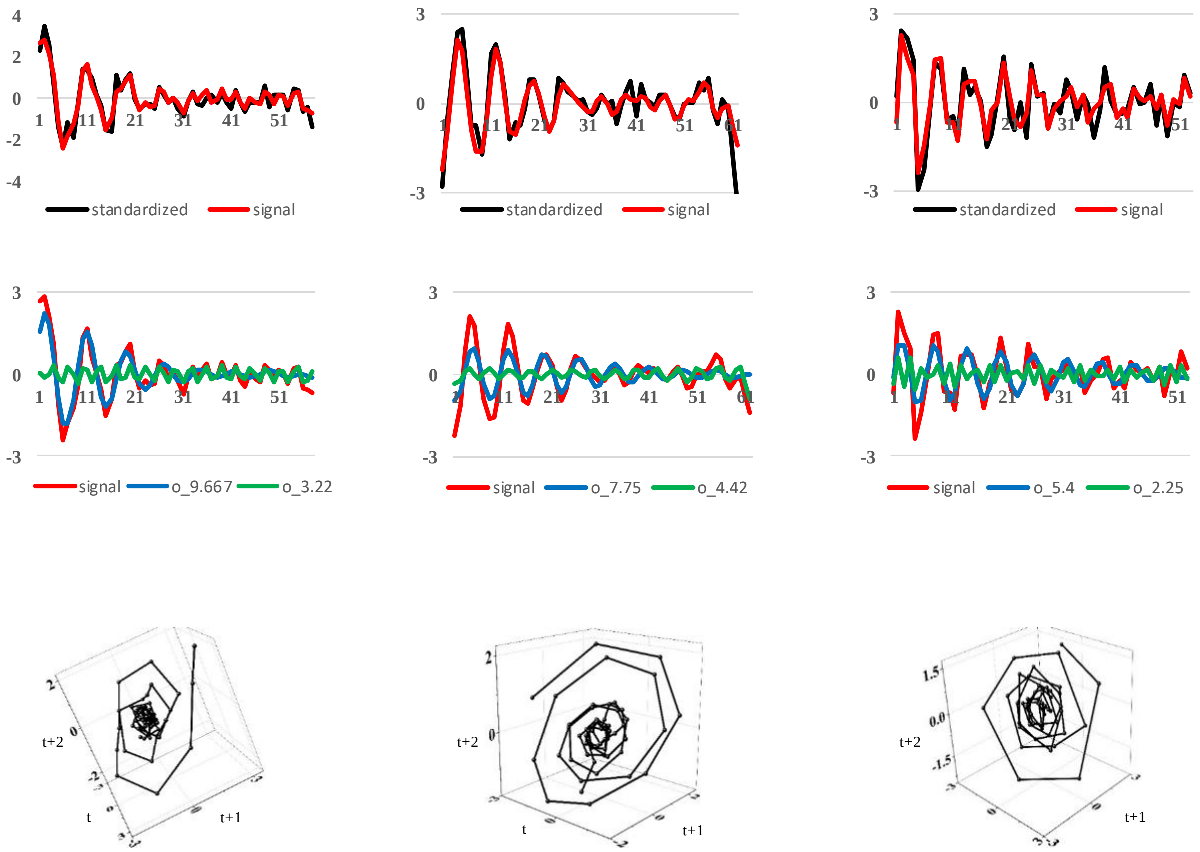

Figure S9. Phase diagrams for cycles with a period ranging from 2 hours to 44 hours.

Decorrelation Times

2nd block

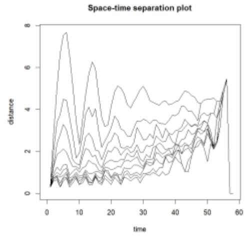

3rd block

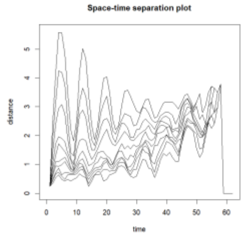

4th block

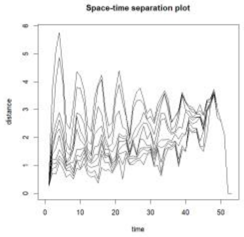

Surrogate Data Tests

2nd block

AAFT	Time Series	surr(low)	surr(high)	H0
Predictive skill	0.704	NA	0.652	Deterministic
Entropy	0.834	0.857	NA	Deterministic
k	20	NA	NA	NA
alpha	0.05	NA	NA	NA

3rd block

AAFT	Time Series	surr(low)	surr(high)	H0
Predictive skill	0.533	NA	0.57	Stochastic
Entropy	0.815	0.85	NA	Deterministic
k	20	NA	NA	NA
alpha	0.05	NA	NA	NA

4th block

AAFT	Time Series	surr(low)	surr(high)	H0
Predictive skill	0.858	NA	0.816	Deterministic
Entropy	0.805	0.927	NA	Deterministic
k	20	NA	NA	NA
alpha	0.05	NA	NA	NA

Figure S10. Fourier transform for cycles with a period ranging from 2 hours to 44 hours.

1.0 mg/L AgNP

A

$$\frac{dx}{dt} = -0.0003 + 0.29z - 0.003y + 0.003yz - 0.11x + 0.0001xz - 0.004xy - 0.0013xyz$$

$$\frac{dy}{dt} = 0.002 - 0.11z + 0.13x - 0.01xz$$

$$\frac{dz}{dt} = 0.0003 + 0.06z - 0.23x - 0.0001xz + 0.002xy$$

$x = O2\_1(t),\; y = pH\_1(t),\; z = O2\_1(t+5)$

B Equilibrium: 0.008, 2.37, 0.03

Eigenvalues: -0.0008, $-0.029 \pm 0.245i$

C

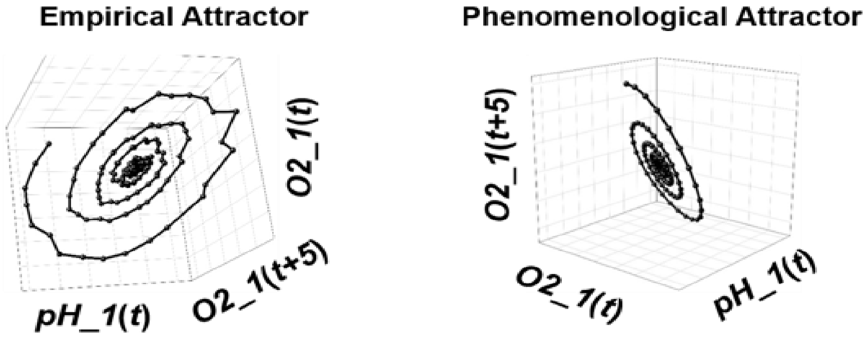

Figure S11. ODEs derived from phenomenological model for 1.0 mg/L AgNP.

2.0 mg/L AgNP

A

$$\frac{dx}{dt} = -0.01 + 0.27z - 0.02y - 0.1x + 0.005xz + 0.006xy + 0.005xyz$$

$$\frac{dy}{dt} = -0.008 + 0.04z + 0.02yz + 0.16x + 0.03xz - 0.02xyz$$

$$\frac{dz}{dt} = 0.01 + 0.05z - 0.01y - 0.023x - 0.0003xz$$

$x = O2\_2(t),\ y = pH\_2(t),\ z = O2\_2(t+5)$

B

Equilibrium: 0.08, -0.4, 0.047

Eigenvalues: -0.002, $-0.021 \pm 0.244i$

C

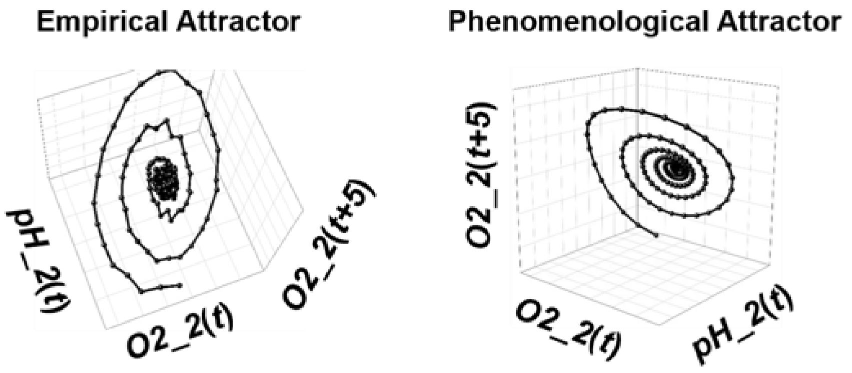

Figure S12. ODEs derived from phenomenological model for 1.0 mg/L AgNP.

3.0 mg/L AgNP

A

$$\frac{dx}{dt} = -0.025 + 0.001w + 0.3z + 0.008zw - 0.006y + 0.001yz + 0.06yzw - 0.14x - 0.003xw + 0.02xz - 0.005xzw + 0.007xyw + 0.002xyz - 0.01xyzw$$

$$\frac{dy}{dt} = 0.016 + 0.43w + 0.03z - 0.04zw - 0.06y - 0.02yz - 0.006yzw$$

$$\frac{dz}{dt} = 0.023 + 0.05z - 0.003zw + 0.005y - 0.001yz - 0.003yzw - 0.21x - 0.012xz + 0.005xzw + 0.002xy - 0.005xyw - 0.003xyz + 0.003xyzw$$

$$\frac{dw}{dt} = -0.022 + 0.011w - 0.38y - 0.04x$$

x = O2_3(t), y = pH_3(t), z = O2_3(t+5), w = pH_3(t+4)

B Equilibrium: $-1, 0.04, -0.38, -0.006$

Eigenvalues: $-0.05, -0.039 \pm 0.217i, 0$

C

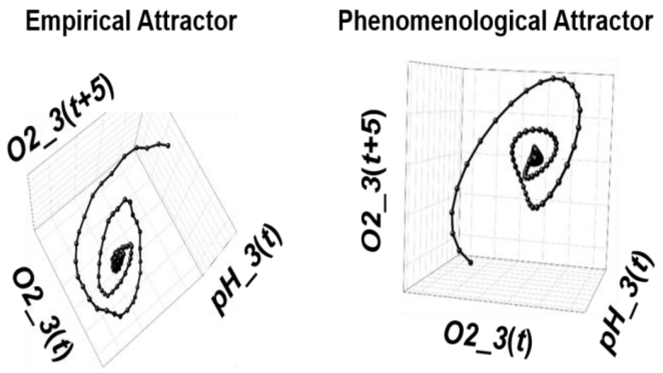

Figure S13. ODEs derived from phenomenological model for 1.0 mg/L AgNP

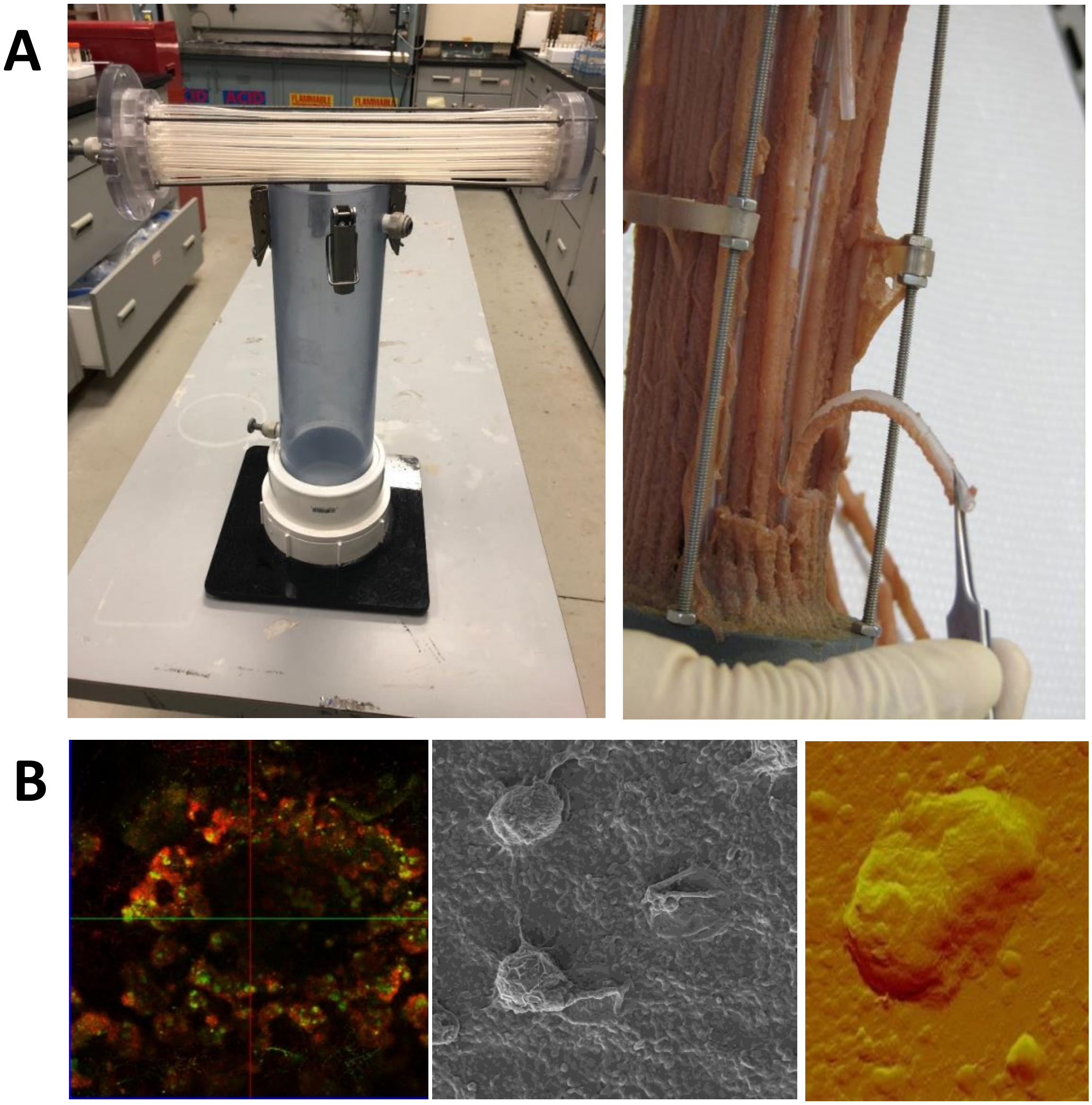

Figure S14. **A)** Photographs of the HfMBR (image on right courtesy of Jay Garland, Space Life Sciences Lab). Details for reactor design and construction may be found in McLamore et al (2008). **B)** Representative confocal microscopy image with live dead stain (left), ESEM (middle) and AFM (right) image of biofilm.

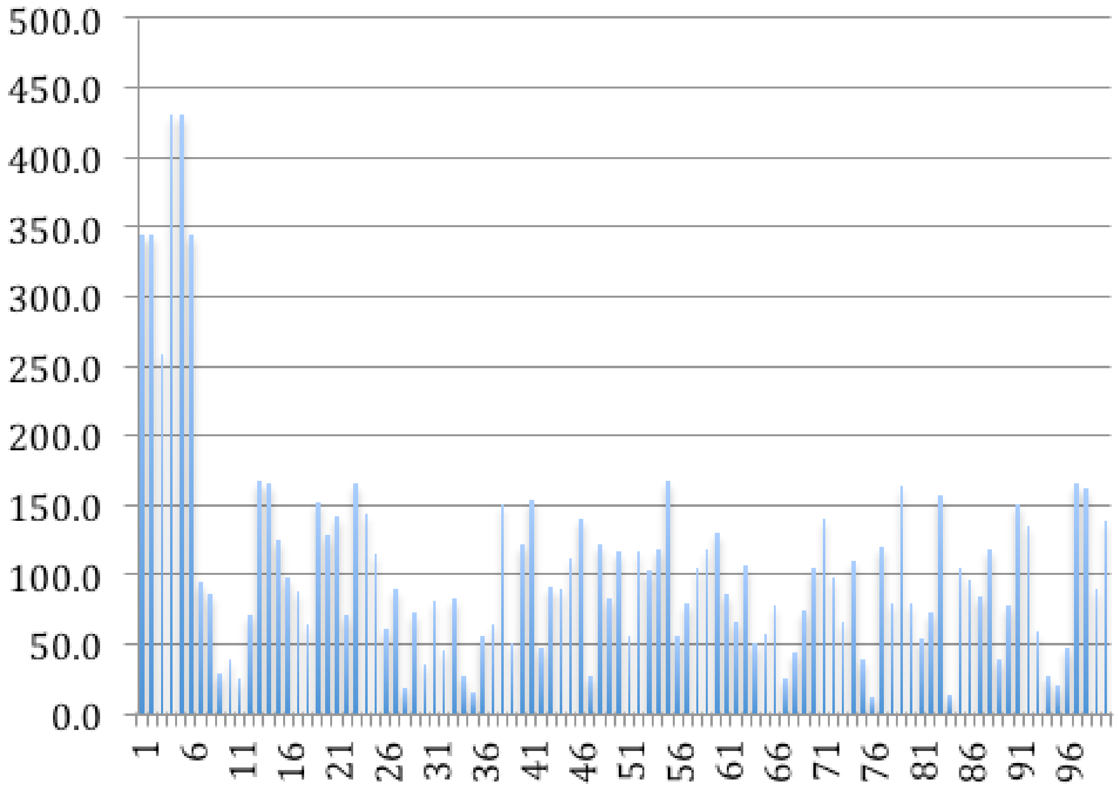

Figure S15. Size distribution of AgNP mixture. Zeta potential and size distribution were measured via dynamic light scattering in 5 mM sodium phosphate buffer adjusted to pH 7.4 using a Zetasizer Nano ZS (Malvern Panalytical, Cambridge, UK).

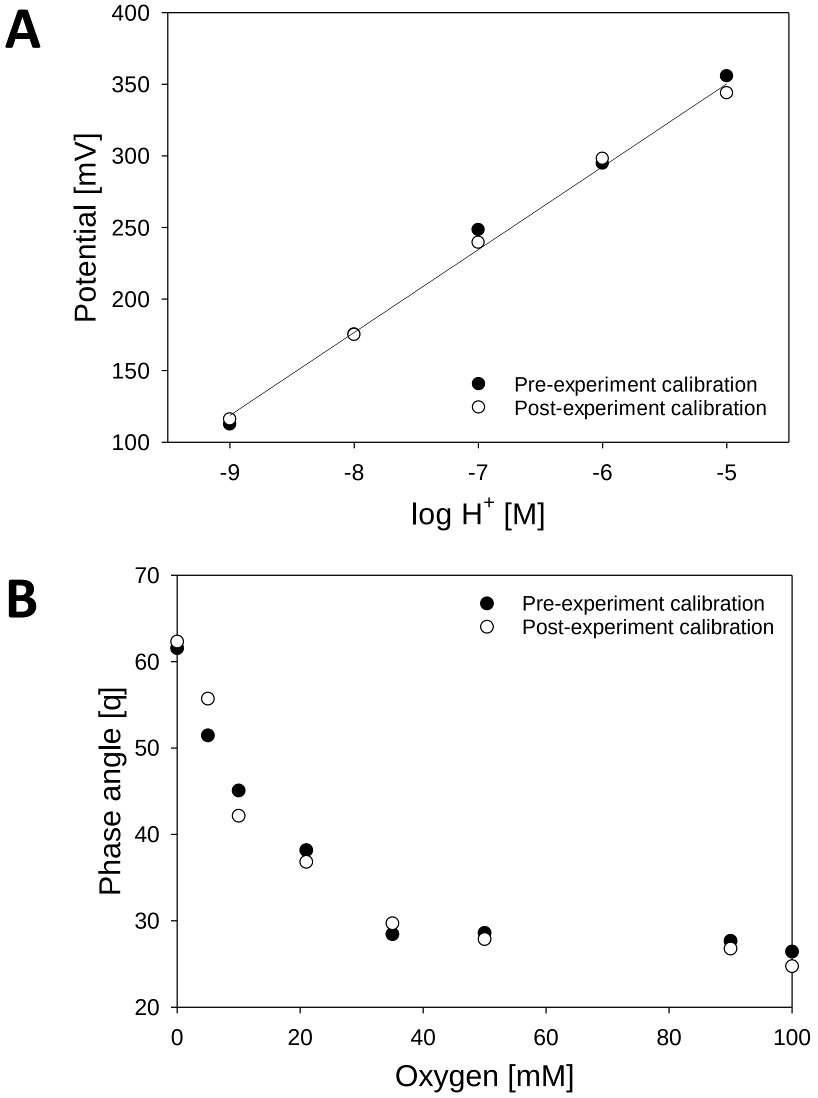

Figure S16. Calibration of **A)** H_+ and **B)** O_2 sensors in GW simulant before and after experimetns. All sensors were calibrated in GW simulant before and after each analysis.

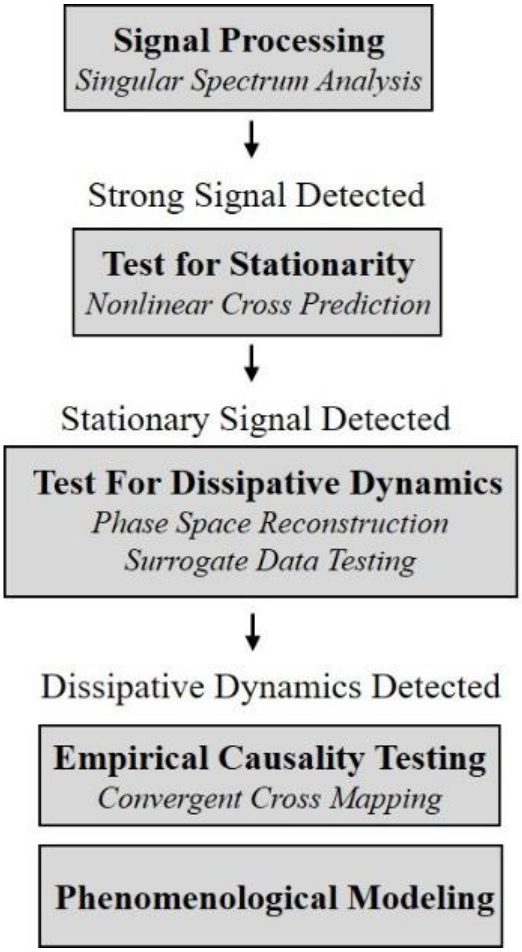

Figure S17. Logic flow for NLTS used in development of phenomenological model. here involved detection, reconstruction, and modeling of bioreactor dynamics from observed output data (pH and O_2).

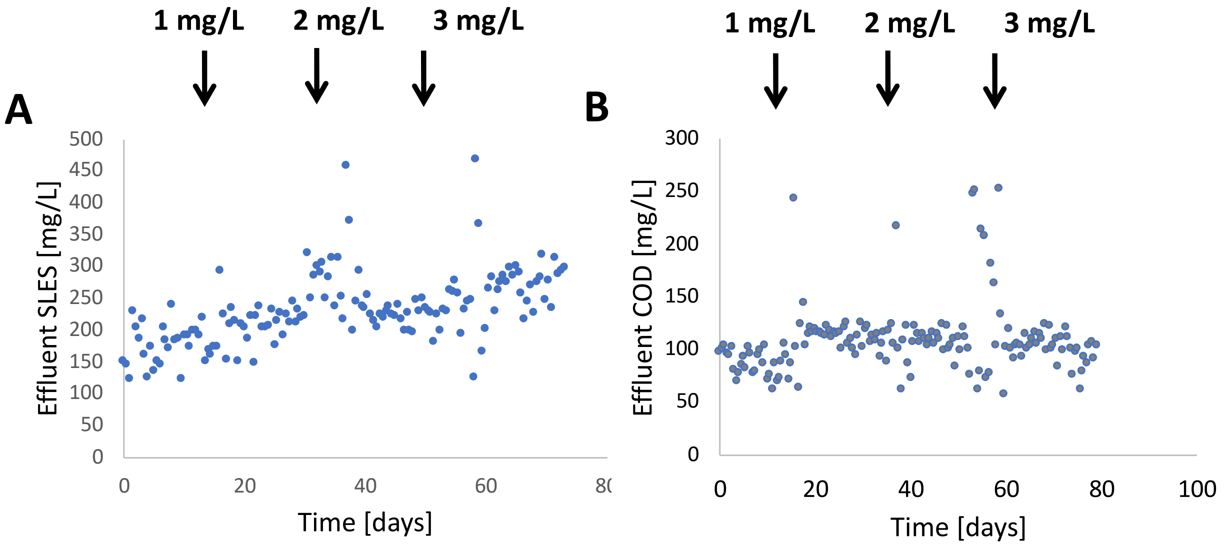

Figure S18. Representative reactor effluent quality for one replicate 5L bench scale HfMBR reactor exposed to cumulative AgNP. A) SLES concentration. B) COD concentration.

Supplemental references:

1 Hamady, M., Walker, J. J., Harris, J. K., Gold, N. J. & Knight, R. Error-correcting barcoded primers for pyrosequencing hundreds of samples in multiplex. *Nat. Methods* **5**, 235-237 (2008).

2 Suzuki, M. T. & Giovannoni, S. J. Bias caused by template annealing in the amplification of mixtures of 16S rRNA genes by PCR. *Appl. Environ. Microbiol.* **62**, 625-630 (1996).
